# The impact of inter-infection time on antimicrobial resistance profiles in women with multiple urinary tract infections over time

**DOI:** 10.1093/jac/dkaf194

**Published:** 2025-06-24

**Authors:** Trenton Honda, Maria D L A Vazquez-Montes, Thomas R Fanshawe, Nicole Stoesser, Christopher C Butler, A Sarah Walker, Gail Hayward

**Affiliations:** Nuffield Department of Primary Care Health Sciences, University of Oxford, Oxford, UK; Nuffield Department of Primary Care Health Sciences, University of Oxford, Oxford, UK; Nuffield Department of Primary Care Health Sciences, University of Oxford, Oxford, UK; Nuffield Department of Medicine, University of Oxford, Oxford, UK; NIHR Health Protection Research Unit in Healthcare Associated Infections and Antimicrobial Resistance, University of Oxford, Oxford, UK; NIHR Oxford Biomedical Research Centre, Oxford University Hospitals NHS Foundation Trust, John Radcliffe Hospital, Oxford, UK; Nuffield Department of Primary Care Health Sciences, University of Oxford, Oxford, UK; Nuffield Department of Medicine, University of Oxford, Oxford, UK; NIHR Health Protection Research Unit in Healthcare Associated Infections and Antimicrobial Resistance, University of Oxford, Oxford, UK; NIHR Oxford Biomedical Research Centre, Oxford University Hospitals NHS Foundation Trust, John Radcliffe Hospital, Oxford, UK; Nuffield Department of Primary Care Health Sciences, University of Oxford, Oxford, UK

## Abstract

**Background:**

Urinary tract infection (UTI) treatment in primary care is increasingly complicated by antimicrobial resistance (AMR), and antimicrobial susceptibility profiles are rarely available to prescribers at the point of prescription. Susceptibility profiles from previous urine culture results could inform prescribing, but little is known about associations between previous and current susceptibilities and the impact of time between infections (inter-infection time) on these associations.

**Methods:**

We analysed routinely collected healthcare records of women ≥16 years in Oxfordshire, UK, who had two or more culture-positive urine specimens consistent with a UTI between 2013 and 2019. We used generalized additive logistic models to estimate associations between resistance to each of eight commonly prescribed antibiotics at first UTI and at second UTI, and their interaction with inter-infection time, adjusted for age and calendar year.

**Results:**

In 10 216 women, significant associations were observed between AMR at first and second UTIs. For all antibiotics, these were largest for short inter-infection times. Pivmecillinam resistance at first UTI (OR: 41.70; 95% CI: 27.70–62.80), followed by fosfomycin (OR: 19.90; CI: 13.66–28.92) and ciprofloxacin resistance (OR: 19.65; 95% CI: 16.30–23.75), were strongly associated with resistance to the same antibiotic at the second UTI for inter-infection times ≤3 months. Lower magnitude associations were observed for other antibiotics. For UTIs caused by *Escherichia coli* only, these associations were generally larger.

**Conclusions:**

In a cohort of women experiencing UTIs, AMR at the first UTI and inter-infection time were key determinants of AMR in the second UTI. This information could inform empirical antimicrobial treatment to limit treatment failure in women with recurrent UTI.

## Introduction

Urinary tract infection (UTI) is the commonest bacterial infection encountered in women in primary care, with approximately half of women experiencing at least one UTI in their lifetime, and 20%–30% having subsequent recurrences.^1–3^  *Escherichia coli* is by far the most frequent causative organism.^4^ Increasingly, UTI treatment has been complicated due to antimicrobial resistance (AMR).^5–7^ Recent studies have identified that recurrent UTI (rUTI), defined as ≥2 UTIs in 6 months or ≥3 in 12 months, is an important risk factor for AMR infections.^8^

There are several potential causes of rUTI, including reinfection from a faecal bacterial reservoir, bladder colonization by organisms that evade treatment, intracellular bacterial reservoirs that can cause reinfection, and/or inadequate antimicrobial treatment (e.g. too short an antibiotic course, inadequate drug levels in the urine, AMR).^9^ Given these mechanisms, it is plausible that causative organisms and/or AMR genes in rUTIs persist from earlier infections. However, important gaps remain in our understanding of the development of AMR in women with rUTI.^10–12^ For example, there is limited prior research examining whether antecedent UTI antibiotic susceptibility patterns predict subsequent AMR; these antecedent susceptibility profile results are generally available and could be useful in guiding empirical treatment at recurrence in the absence of point-of-care antimicrobial susceptibility tests. One Italian study found that having an initial fluoroquinolone-resistant *E. coli* UTI was associated with an 85% increased likelihood of a subsequent fluoroquinolone-resistant *E. coli* UTI, and that the AMR profile of the *E. coli* isolates in the index and rUTIs were the same in 61% of women.^13^ In a US cohort, Cohen *et al.*^14^ identified that prior resistance to trimethoprim/sulfamethoxazole, nitrofurantoin or ciprofloxacin was the strongest predictor of resistance to the same antibiotic in subsequent UTI episodes, indicating that prior culture results in women experiencing multiple UTIs are important predictors of subsequent AMR. However, the magnitude of the association between baseline and subsequent susceptibility profiles, and whether this relationship is confounded by age, remains unclear.

Additionally, although rUTI is defined based upon the timeframes between multiple UTI episodes, very little prior literature directly explores the impact of inter-infection time (i.e. time between two UTI episodes) on antibiotic susceptibility profiles.^15–17^ The temporally dependent definition of rUTI is arbitrary and without specific biological foundation. It is possible that dichotomizing infections as meeting or not the formal rUTI definition loses clinically relevant information. The impact of inter-infection time on AMR, how this interval modifies the effect of antibiotic susceptibilities of the prior UTI (statistical interaction), and whether the associations differ by antibiotic class, has clinical relevance, as it could help personalize empirical rUTI treatment.

We therefore investigated how AMR profiles in antecedent infections affect the likelihood of AMR in subsequent infections, and to what extent inter-infection time modifies these associations for all bacterial species and for infections caused by *E. coli* only, in a population of women in Oxfordshire, UK.

## Methods

### Population

We conducted an electronic health records study of women aged ≥16 years in the Infections in Oxfordshire Research Database (IORD). IORD is a de-identified database that has primary and secondary care urine culture microbiological data linked to patient demographic and clinical records, and Research Ethics Committee and Confidentiality Advisory Group approval for research without individual patient consent (19/SC/0403, 19/CAG/0144).^18^ Consistent with our focus on community-acquired UTIs (Figure S1, available as Supplementary data at *JAC* Online), we included only urine cultures from primary care settings and cultures requested within 48 h of hospital admission. We excluded: urine cultures from participants with records of inpatient hospitalization within Oxfordshire within 28 days prior to the urine collection; patients with evidence of mislinkage (e.g. a linked date of death before the study period, cultures with collection dates >48 h after date of death); cultures explicitly requested for antenatal screening and test results dated >24 h before the time the specimen was collected; samples not identified as midstream or clean-catch urine samples (i.e. catheter samples); samples outside of the study period 1 June 2013 to 31 December 2019; samples without a pure or predominant growth of bacteria in urine culture at ≥10^4^ cfu/mL; cultures without microbiology results or where the test failed. Last, we restricted our study to women with two or more culture-positive urine samples consistent with UTI, with each eligible UTI episode ≥14 days from any prior UTI episode to increase the likelihood that the episodes were distinct infections.

### Analysis

Primary analyses included the first and second sequential UTI episodes during the study period attributed to any uropathogen; secondary analyses included the first and second UTI episodes caused by *E. coli*. For example, if participant ‘X’ had three UTI infections, the first and third caused by *E. coli*, and the second caused by another uropathogen, our primary analysis included the first and second infection, whereas our secondary analysis included the first and third infections. Information from later UTI episodes was not used. We considered susceptibility results for: amoxicillin, amoxicillin/clavulanate (co-amoxiclav), cefalexin, ciprofloxacin, fosfomycin, nitrofurantoin, pivmecillinam and trimethoprim as these are the commonest antibiotics prescribed in primary care in the UK. The <0.7% of results reported as intermediate susceptibility were grouped with resistant results. We categorized the inter-infection times for each woman as 0 to <3 months, 3 to <6 months, 6 to <12 months, or ≥12 months based on arbitrary but pragmatic time windows physicians might use when making empirical prescribing decisions. We then modelled the odds of resistance at the second infection to each individual antibiotic of interest as a function of a multiplicative interaction between the binary resistance status (resistant versus susceptible) of the same antibiotic in the first infection, and the four-level categorical inter-infection time variable using multivariable logistic regression in complete cases. We fitted eight models, one for each antibiotic of interest. All models were adjusted for participant age, a key risk factor, and calendar year. We used age at second infection, as this would be more obvious to physicians when evaluating the second infection. Calendar year was adjusted to account for longitudinal AMR trends.

We then used generalized additive models (GAMs) with logistic link and penalized regression splines to estimate predicted probabilities of AMR at the second infection given AMR at the first infection for each individual antibiotic across continuous inter-infection times between 0 and 36 months.^19^ Continuous age at second infection was controlled for using a penalized spline function. Extreme inter-infection times >36 months (approximately the 96th percentile) were truncated to 36 months to reduce influence of outliers. Predicted probabilities were estimated for first infections that were resistant or susceptible to each antibiotic, at the median age (68 years) and calendar year (2016). For all splines, the number of basis functions was selected using the Akaike Information Criteria.^20,21^

We repeated the multivariable logistic regression and GAM analyses for UTI pairs where the causative organisms were *E. coli* for both infections. Secondly, for both populations, we repeated the multiple logistic regression and GAM analyses including all pre-specified antibiotics [other than pivmecillinam and fosfomycin where numbers of resistant organisms and number of cultures reporting sensitivity results were relatively low (Table 1) which affected convergence] and their interactions with inter-infection time in the same model. Predictions were estimated at the median age and calendar year (as above), and all other antibiotics controlled for in the model as ‘susceptible’. Sensitivity analyses were undertaken as follows: categorized intermediate susceptibility results as susceptible or excluded intermediate susceptibility results, examined GAM prediction models at all other calendar years (2013–2019), stratified models above and below age 50, and examined an alternative definition of each eligible UTI episode as ≥28 days from any prior UTI episode.

**Table 1.** Urine culture antimicrobial resistance prevalence in first and second UTIs for eight antibiotics commonly used in primary care

	First UTI: % resistant (*n* resistant/total cultures)^a^	Second UTI: % resistant (*n* resistant/total cultures)^a^
Amoxicillin	48.6	49.0
	(4845/9978)	(4865/9927)
Co-amoxiclav	27.6	28.6
	(2580/9339)	(2607/9104)
Cefalexin	21.3	19.5
	(1847/8665)	(1655/8508)
Ciprofloxacin	14.7	16.5
	(1483/10 086)	(1652/9982)
Fosfomycin	4.4	4.2
	(395/8932)	(371/8752)
Nitrofurantoin	9.1	9.3
	(912/10 006)	(927/9929)
Pivmecillinam	10.5	10.8
	(360/3427)	(487/4498)
Trimethoprim	32.6	36.2
	(3279/10 051)	(3619/9985)

^a^ *n* = number of cultures: this varies as not all cultures were tested for all antibiotics (e.g. pivmecillinam was rarely tested before 2016).

## Results

A total of 11 881 women had at least two culture-positive urine samples consistent with UTI in the initial data extract (Figure S1). After restricting to only urine cultures representing likely community-acquired infections, mid-stream or clean catch samples, and cultures that occurred between 1 June 2013 and 31 December 2019, the final study population included 10 216 women. AMR prevalence at the first UTI episode in the study period was highest for amoxicillin, with 48.6% of first infections resistant, followed by trimethoprim (32.6%) and co-amoxiclav (27.6%). The lowest rates of resistance were observed for nitrofurantoin (9.1%) and fosfomycin (4.4%; Table 1). At the second infection, the median (IQR) age was 68 (47–79) years. Participant ages were somewhat bimodally distributed, with ages aggregating around the 16–40 and 60–80 year ranges (Figure S2). Inter-infection times were significantly right skewed, with a median (IQR) inter-infection time of 3.8 (1.5–9.7) months and a range of 0.5–65.8 months (Figure S3).

In our single-antibiotic logistic regression models (Table 2), the strongest associations with AMR for all antibiotics were observed for inter-infection times 0 to <3 months, with monotonic decreases in association as inter-infection times increased, and the smallest associations with AMR for all antibiotics for inter-infection times ≥12 months. The magnitudes of association varied significantly across antibiotics. For example, pivmecillinam resistance in the first UTI was associated with 41.70 higher odds (95% CI: 27.70–62.80) of pivmecillinam resistance in the second UTI amongst participants with inter-infection times <3 months, followed by fosfomycin (OR: 19.90; 95% CI: 13.66–28.92) and ciprofloxacin (OR: 19.65, 95% CI: 16.30–23.75). Lower magnitude but strong associations were observed for all other antibiotics, and these associations remained statistically significant for inter-infection times ≥12 months. Older age was associated with somewhat higher resistance at second infection for some antibiotics (i.e. amoxicillin, co-amoxiclav, cefalexin, fosfomycin, nitrofurantoin) but not for others (i.e. ciprofloxacin, pivmecillinam, trimethoprim) in these eight single-antibiotic models (Table S1). There was some evidence of decreasing AMR over time for amoxicillin and cefalexin, and trimethoprim for *E. coli*, whereas ciprofloxacin resistance increased over time (Table S2).

**Table 2.** Association between antimicrobial resistance at first and second infection by inter-infection time for all bacterial species for eight antibiotics commonly used in primary care^a^

	0 to <3 mo	3 to <6 mo	6 to <12 mo	≥12 mo
	OR (95% CI)	OR (95% CI)	OR (95% CI)	OR (95% CI)
Amoxicillin	12.38	6.55	4.85	1.84
	(10.76, 14.25)	(5.40, 7.94)	(3.78, 6.23)	(1.54, 2.20)
	*P* < 0.001	*P* < 0.001	*P* < 0.001	*P* < 0.001
Co-amoxiclav	10.64	7.12	4.32	2.09
	(9.06, 12.49)	(5.65, 8.97)	(3.21, 5.82)	(1.67, 2.63)
	*P* < 0.001	*P* < 0.001	*P* < 0.001	*P* < 0.001
Cefalexin	9.75	6.68	4.07	2.02
	(8.04, 11.82)	(4.99, 8.96)	(2.80, 5.90)	(1.52, 2.69)
	*P* < 0.001	*P* < 0.001	*P* < 0.001	*P* < 0.001
Ciprofloxacin	19.65	9.44	7.93	3.61
	(16.30, 23.75)	(7.15, 12.50)	(5.45, 11.54)	(2.66, 4.89)
	*P* < 0.001	*P* < 0.001	*P* < 0.001	*P* < 0.001
Fosfomycin	19.90	13.80	14.50	2.50
	(13.66, 28.92)	(7.60, 25.10)	(6.03, 34.80)	(1.16, 5.42)
	*P* < 0.001	*P* < 0.001	*P* < 0.001	*P* = 0.020
Nitrofurantoin	15.04	6.30	5.77	3.76
	(11.91, 19.00)	(4.30, 9.22)	(3.60, 9.24)	(2.55, 5.54)
	*P* < 0.001	*P* < 0.001	*P* < 0.001	*P* < 0.001
Pivmecillinam	41.70	16.47	8.99	4.73
	(27.70, 62.80)	(8.56, 31.68)	(3.41, 23.70)	(2.02, 11.04)
	*P* < 0.001	*P* < 0.001	*P* < 0.001	*P* < 0.001
Trimethoprim	10.94	5.94	4.63	2.25
	(9.48, 12.61)	(4.86, 7.27)	(3.54, 6.04)	(1.85, 2.75)
	*P* < 0.001	*P* < 0.001	*P* < 0.001	*P* < 0.001

^a^All (single-antibiotic) models control for calendar year at second infection, and age at second infection. There is strong evidence of heterogeneity across time periods for all antibiotics; see Figure 1.

**Figure 1.**
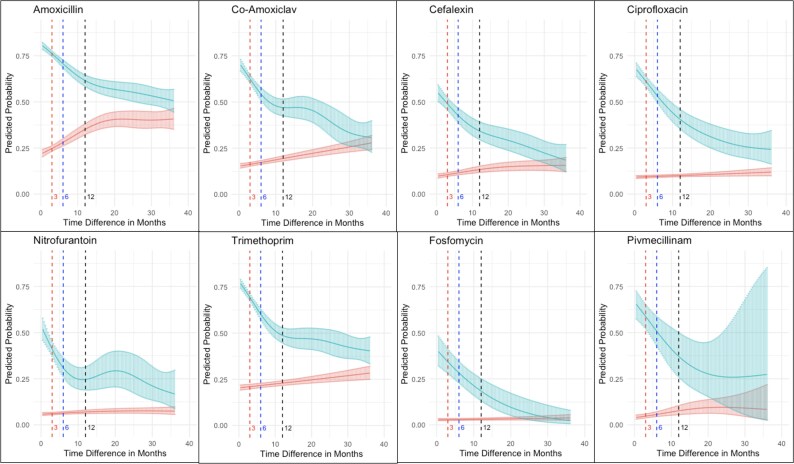
Predicted probabilities of antimicrobial resistance at the second UTI given resistance (blue) or susceptibility (red) to the same antibiotic at the first UTI (single-antibiotic models). Models control for calendar year at second infection, and age at second infection with predictions made in 2016 and age 68 years. Red, blue and black vertical dashed lines show 3, 6 and 12 month predicted probabilities, respectively, corresponding to the thresholds in the categorical model in Table 2. Solid blue/red curves show point estimates and shaded areas around the point estimates show 95% confidence intervals.

Our GAM models (Figure 1) showed that for resistance at first UTI, the probability of resistance at the second UTI dropped rapidly but continuously for inter-infection times over the first year, then remained relatively stable or continued to decrease at a slower rate. For example, for the shortest inter-infection times (14 days), predicted probabilities of resistance at second UTI given resistance at the first UTI varied from 0.84 (amoxicillin) to 0.40 (fosfomycin); by 1 year these had dropped to 0.61 (amoxicillin) and 0.18 (fosfomycin). Variability was also observed in the absolute predicted probability of resistance at the second UTI given susceptibility at the first UTI, from 0.03 (fosfomycin) to 0.22 (amoxicillin), and this generally increased with increasing inter-infection time, albeit to a much smaller extent than the decreases observed for resistant first UTI. Consequently, predicted probabilities for resistance at second UTI given resistance (blue in Figure 1) or susceptibility (red) at first UTI converged by 36 months for most, but not all (e.g. ciprofloxacin, trimethoprim) antibiotics.

**Figure 2.**
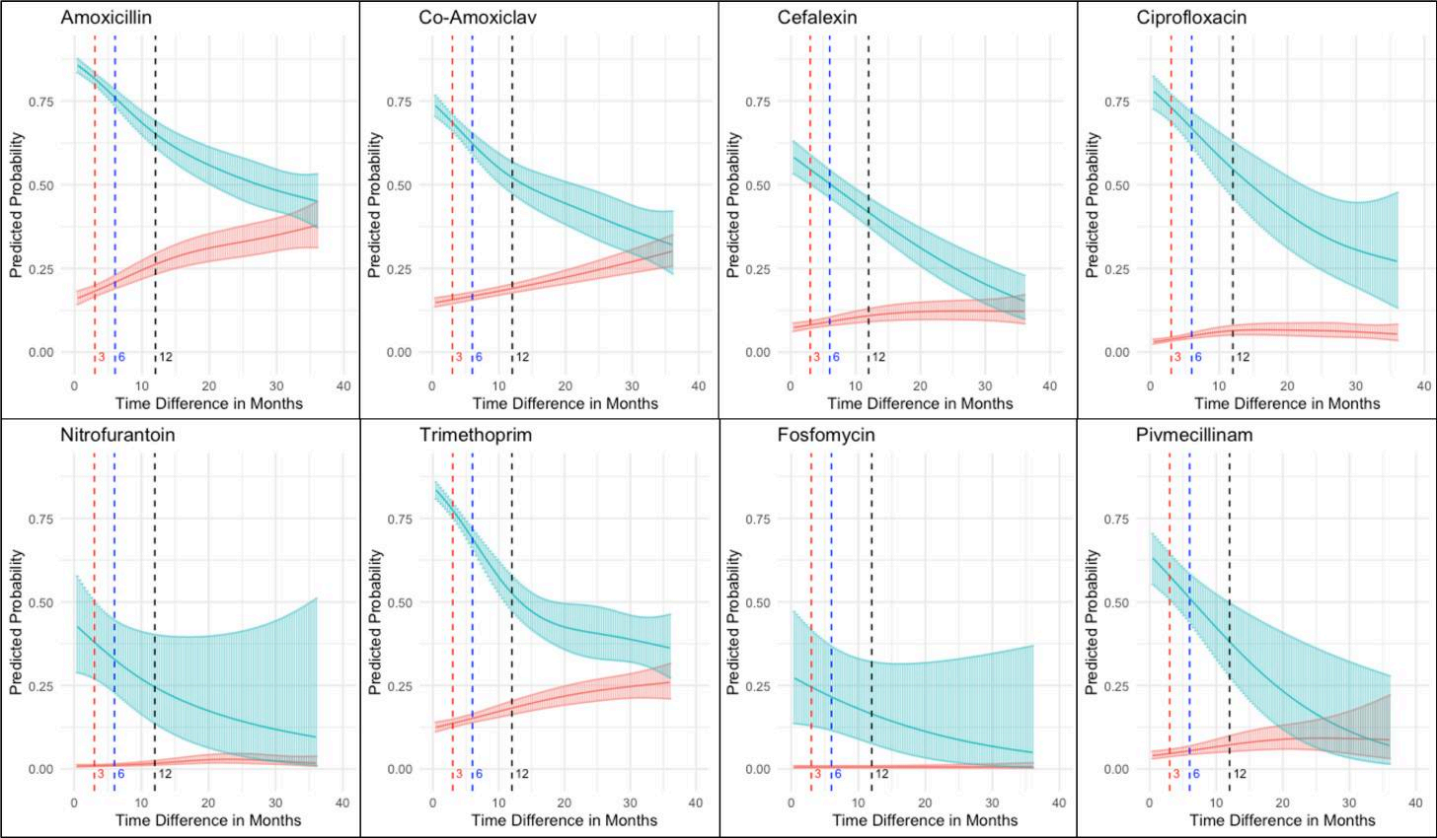
Predicted probabilities of antimicrobial resistance at the second UTI given resistance (blue) or susceptibility (red) to the same antibiotic at the first UTI (single-antibiotic models) in UTI pairs caused by *E. coli*. Models control for calendar year at second infection, and age at second infection with predictions made in 2016 and age 68 years. Red, blue and black vertical dashed lines show 3, 6 and 12 month predicted probabilities, respectively, corresponding to the thresholds in the categorical model in Table 3. Solid blue/red curves show point estimates and shaded areas around the point estimates show 95% confidence intervals.

For UTI pairs where the causative organisms were *E. coli* for both infections, associations in our single-antibiotic logistic regression models were larger in magnitude compared with those observed for all bacterial species for most antibiotics and inter-infection windows (Table 3). For example, associations in the 0 to <3 month inter-infection window were 2.2 times larger for amoxicillin (OR *E. coli*: 27.35 versus 12.38 all species), 5.3 times larger for ciprofloxacin (OR *E. coli*: 104.23 versus 19.65 all species), 3.1 times larger for fosfomycin (OR *E. coli*: 61.34 versus 19.90 all species), 6.1 times larger for nitrofurantoin (OR *E. coli*: 91.46 versus 15.04 all species) and 2.8 times larger for trimethoprim (OR *E. coli*: 30.17 versus 10.94 all species). Associations were also higher for co-amoxiclav (OR *E. coli*: 14.21 versus 10.64 all species), and cefalexin (OR *E. coli*: 15.73 versus 9.75 all species), albeit less markedly so. Pivmecillinam was the only antibiotic for which associations were nominally lower (OR *E. coli*: 40.39 versus 41.70 all species). Associations decreased in a generally monotonic fashion as inter-infection times increased (Table 3). For all antibiotics, associations remained statistically significant for inter-infection times ≥12 months. In our GAM models for UTI pairs where the causative organisms were *E. coli* for both infections (Figure 2), the general patterns of association were similar to models including all uropathogens, but the smaller sample sizes generally produced wider CIs as inter-infection times increased.

**Table 3.** Association between antimicrobial resistance at first and second infection by inter-infection time in UTI pairs caused by *E. coli* for eight antibiotics commonly used in primary care^a^

	0 to <3 mo	3 to <6 mo	6 to <12 mo	≥12 mo
	OR (95% CI)	OR (95% CI)	OR (95% CI)	OR (95% CI)
Amoxicillin	27.35	12.43	8.12	2.41
	(22.38, 33.41)	(9.57, 16.13)	(5.81, 11.35)	(1.90, 3.05)
	*P* < 0.001	*P* < 0.001	*P* < 0.001	*P* < 0.001
Co-amoxiclav	14.21	10.00	5.59	2.18
	(11.71, 17.26)	(7.60, 13.15)	(3.92, 7.99)	(1.66, 2.85)
	*P* < 0.001	*P* < 0.001	*P* < 0.001	*P* < 0.001
Cefalexin	15.73	12.96	5.84	2.91
	(12.25, 20.20)	(8.90, 18.87)	(3.64, 9.37)	(1.99, 4.23)
	*P* < 0.001	*P* < 0.001	*P* < 0.001	*P* < 0.001
Ciprofloxacin	104.23	34.23	28.09	10.49
	(72.82, 149.19)	(21.75, 53.86)	(15.02, 52.54)	(6.25, 17.61)
	*P* < 0.001	*P* < 0.001	*P* < 0.001	*P* < 0.001
Fosfomycin	61.34	38.39	56.40	27.26
	(24.08, 156.21)	(8.81, 167.32)	(8.48, 374.98)	(6.49, 114.43)
	*P* < 0.001	*P* < 0.001	*P* < 0.001	*P* < 0.001
Nitrofurantoin	91.46	24.63	32.16	9.72
	(44.73, 187.02)	(6.96, 87.14)	(7.05, 146.64)	(2.55, 37.02)
	*P* < 0.001	*P* < 0.001	*P* < 0.001	*P* = 0.001
Pivmecillinam	40.39	16.41	8.98	4.40
	(26.68, 61.15)	(8.53, 31.37)	(3.41, 23.68)	(1.83, 10.58)
	*P* < 0.001	*P* < 0.001	*P* < 0.001	*P* = 0.001
Trimethoprim	30.17	13.37	8.90	2.38
	(24.35, 37.38)	(10.09, 17.73)	(6.15, 12.88)	(1.81, 3.13)
	*P* < 0.001	*P* < 0.001	*P* < 0.001	*P* < 0.001

^a^All (single-antibiotic) models control for calendar year at second infection and age at second infection. There is strong evidence of heterogeneity across time periods for all antibiotics; see Figure 2.

We also undertook multi-antibiotic analyses modelling the associations between AMR at first UTI to six antibiotics of interest simultaneously (excluding pivmecillinam and fosfomycin as the numbers of resistant organisms and number of cultures reporting sensitivity results were low; Table 1). Models assessed associations between AMR to six antibiotics at the first infection (and their interactions with inter-infection time as covariates in the same model) and AMR at the second UTI, for amoxicillin (Table S3, Figure S4), co-amoxiclav (Table S4, Figure S5), cefalexin (Table S5, Figure S6), ciprofloxacin (Table S6, Figure S7), nitrofurantoin (Table S7, Figure S8) and trimethoprim (Table S8, Figure S9) (Tables S9–S14 and Figures S10–S15 show the same analyses in UTI pairs caused by *E. coli*). In general, resistance at the second UTI was significantly associated with resistance to the same antibiotic at the first UTI, although associations were somewhat attenuated compared with the single-antibiotic models, most prominently for co-amoxiclav, cefalexin and ciprofloxacin. There was generally (although not exclusively) no evidence of association with first-UTI resistance to the other antibiotics included in the multi-antibiotic models. The most notable exception was amoxicillin resistance at the first UTI, which was independently associated with co-amoxiclav resistance at second UTI, as was co-amoxiclav resistance at the first UTI. Effects of age and calendar time were consistent with our single-antibiotic models (Tables S1 and S2).

Sensitivity analyses recategorizing the small percentage of cultures reported as intermediate resistance as susceptible or excluding them from the analysis entirely gave similar results. Additionally, given the small magnitude of effects (Table S2) changing the calendar year in our prediction models did not importantly change the predicted probabilities (data not shown). In models stratified by age (Table S15), there were several antibiotics for which magnitudes of association were stronger for those <50 years old compared with older individuals (i.e. amoxicillin, co-amoxiclav, cefalexin, fosfomycin, nitrofurantoin and pivmecillinam), whereas associations were only significantly stronger in those ≥50 years for trimethoprim. Last, when using an alternative definition of each eligible UTI episode as ≥28 days from any prior UTI episode, associations were mildly attenuated, but did not importantly alter our results (Table S16).

## Discussion

We identified strong, statistically significant associations between resistance at the second UTI and resistance to the same antibiotic at first UTI, among women with UTI recurrence. The strength of associations for all antibiotics varied by inter-infection time, with the largest magnitude associations observed for the shortest inter-infection times, and generally monotonic decrements in association as inter-infection times increased to ≥12 months. We also observed that susceptibility to all antibiotics at first UTI was associated with low predicted probabilities of resistance at the second UTI, particularly for the shortest inter-infection times. As inter-infection times increased, the AMR profile at first UTI became less informative but there were still substantial differences at 12 months, the maximum current threshold used to define a recurrent UTI. These findings were true for all bacterial species and *E. coli* considered separately.

When evaluating women who experience UTIs in primary care, prescribers often have information on the timing and antimicrobial susceptibility profile of antecedent UTIs. We found that these two variables were strong predictors of AMR in subsequent UTIs and can therefore be used to guide empirical antimicrobial therapy. A second UTI within 2–3 weeks of the first UTI was associated with 60%–80% probability of resistance to the same antibiotic(s) in the first UTI and only 0%–20% probability of resistance if susceptible to (an) antibiotic(s) at the first UTI, although at 36 months these effects were appreciably attenuated. However, our results suggest that significant and important AMR may persist for over a year after the first UTI.

Our findings are broadly consistent with recent studies predicting AMR in rUTIs. For example, a recent US study found that trimethoprim resistance was strongly associated with trimethoprim-resistant bacteria in antecedent UTIs with a predicted resistance rate of ∼65%.^22^ In a Korean cohort (*n* = 180 women) 71% and 68% of participants with an index ESBL-producing *E. coli* UTI had ESBL identified in their first and second recurrent infections, respectively.^15^ Inter-infection time was a strong predictor of recurrence, with those with ESBL-associated recurrences having a mean inter-infection time of 3.2 months (SD ±3.6), whereas those without ESBL at subsequent infections had a mean inter-infection time of 9.8 months (±12.4).^15^ These studies were, however, limited in focusing on single antimicrobial resistances. In a study of 80 267 US women with rUTI, participants had an 18% increased odds of single-drug AMR and up to 70% increased odds for MDR in the second UTI relative to women without rUTI.^23^ In an inpatient population in Israel, longer inter-infection times were associated with lower odds of resistance at subsequent infection for ciprofloxacin-resistant Gram-negative bacteria (OR per day 0.999; 95% CI: 0.999–1.000; *P* < 0.001), ESBL-producing Enterobacteriaceae (OR per day 0.999; 95% CI: 0.999–0.999; *P* < 0.001) and carbapenem-resistant non-fermenters (OR per day 0.998; 95% CI: 0.996–1.000; *P* = 0.032), but not carbapenem-resistant Enterobacteriaceae.^17^ Consistent with our work, another study in Israel also observed that paired community-acquired infections >1 week apart displayed high concordance of AMR profiles, with risk ratios (RRs) of resistance at second infection given resistance at the first infection decaying over 112 weeks for trimethoprim/sulfamethoxazole (RR: ∼4 to 1.5), ciprofloxacin (RR: ∼4 to 2), co-amoxiclav and cefalexin (RR: ∼8 to 2.5), and nitrofurantoin (RR: ∼9 to 3).^16^ Our findings add to the understanding of AMR in UTI in other geographical settings,^14^ the simultaneous impact of several antibiotic susceptibility profiles in multi-antibiotic models, and have considered the largest number of commonly prescribed antibiotics to date.

Our study has several limitations. First, although the cohort is large, it is restricted to one (albeit large) geographical region of the UK and the time period immediately pre-COVID; AMR epidemiology may vary by geography and timeframe. Second, the dataset only reflects samples sent for testing, missing UTIs that were treated without culturing, that were reported as mixed growth or did not meet reportable thresholds for infection, which may introduce selection bias into the analysis. Lastly, we could not evaluate potentially important confounders, including antibiotic use and health behaviours, which may affect the risk of developing multiple UTIs and/or AMR as these were not available in IORD.

Overall, in a cohort of women experiencing multiple UTIs over time in Oxfordshire, UK, the antibiotic susceptibility profile at the first UTI and the time between first and second UTI were highly predictive of AMR in the second UTI. Associations were strong and consistent across all commonly used antibiotic classes, with the strongest associations observed for the shortest inter-infection times. This information could help guide empirical antimicrobial treatment to minimize treatment failure in women experiencing multiple UTIs over time and provides new insights into how the inter-infection time can be applied in clinical decision-making.

## Supplementary Material

dkaf194_Supplementary_Data

## Supplementary data

Figures S1 to S15 and Tables S1 to S16 are available as Supplementary data at *JAC* Online.
