# Supplementary material for: The impact of inter-infection time on antimicrobial resistance profiles in women with multiple urinary tract infections over time
Source: J Antimicrob Chemother. 2025 Jun 24;80(8):2234–40. doi: 10.1093/jac/dkaf194 (PMC12313461; doi:10.1093/jac/dkaf194)
Supplement: dkaf194_Supplementary_Data [file dkaf194_supplementary_data.docx]

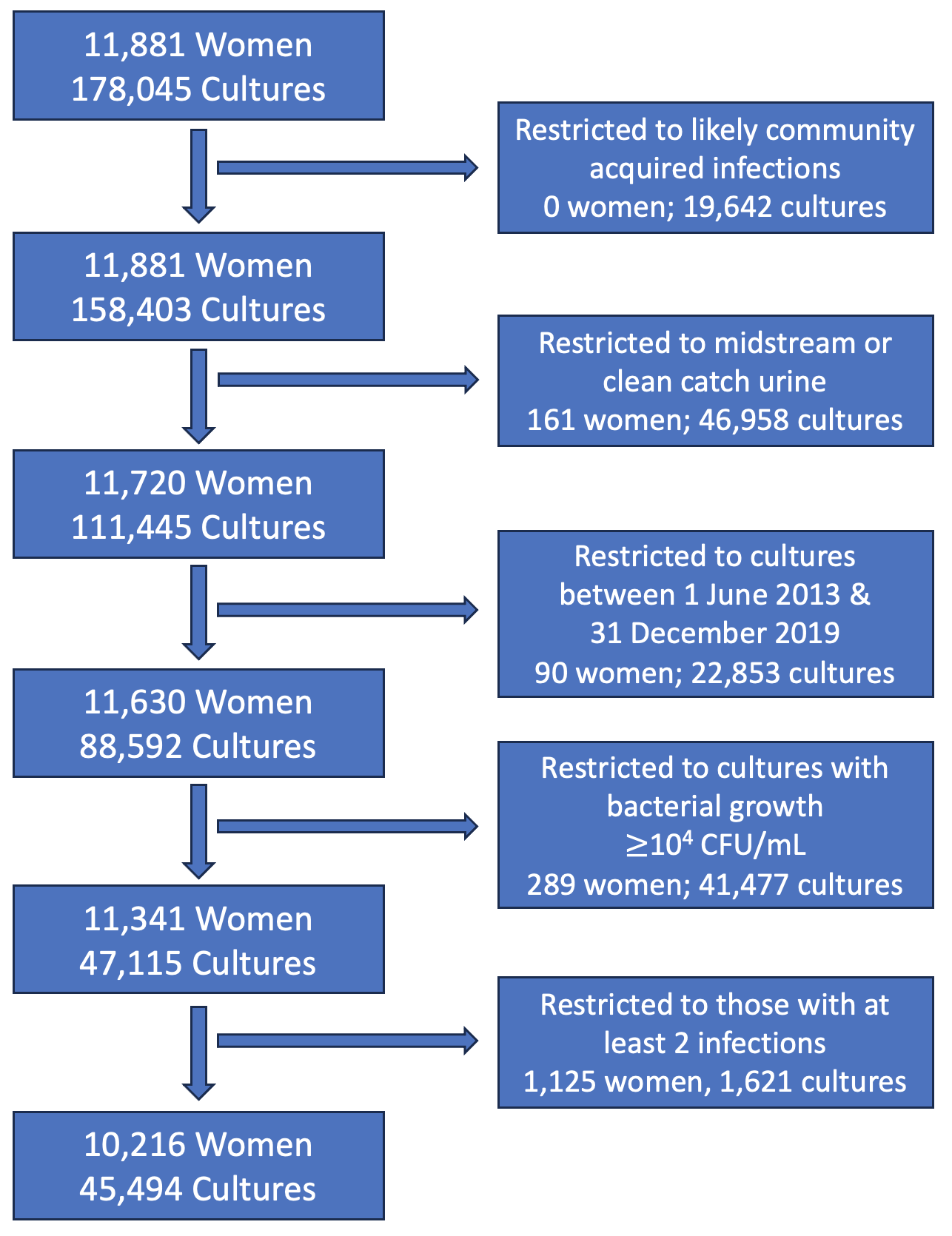


**Figure S1.** Study population flowchart and inclusion and exclusion criteria


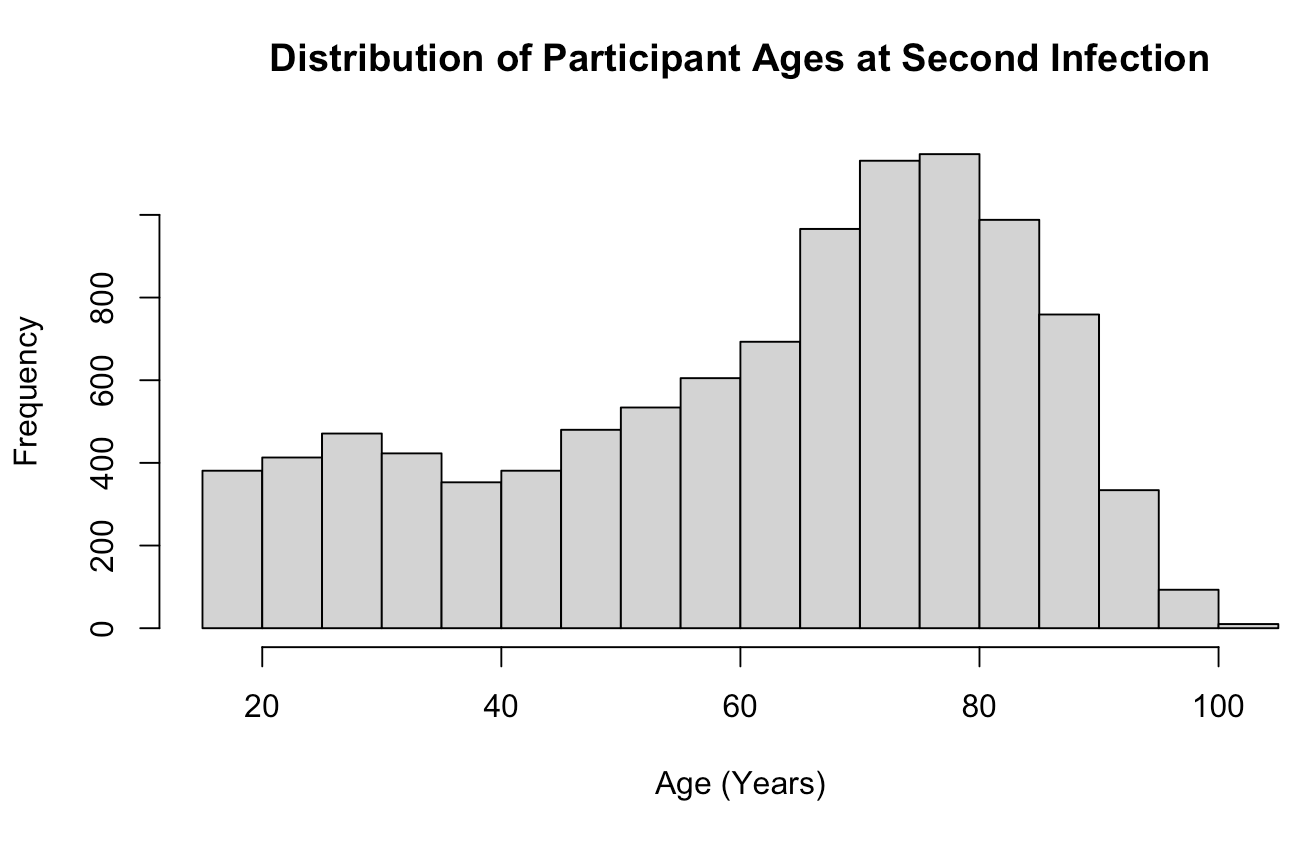


**Figure S2.** Distribution of participant ages


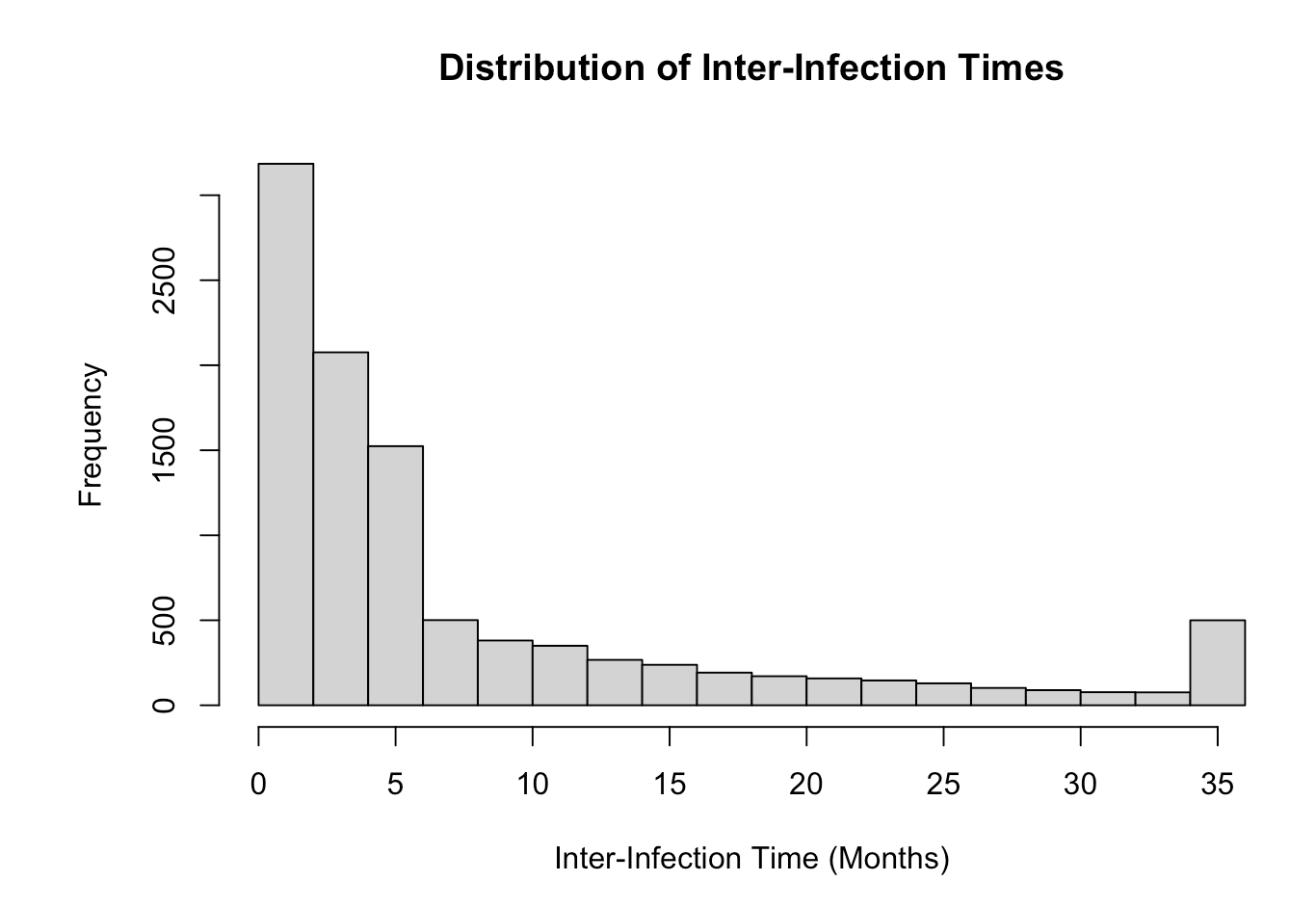


**Figure S3.** Distribution of inter-infection times (months)

**Figure S4:** Predicted probability models of **amoxicillin** resistance at second infection, given resistance (blue) or susceptibility (red) to the indicated antibiotic at first infection, by inter-infection time, controlling for all antibiotic susceptibilities at first infection (multi-antibiotic models)


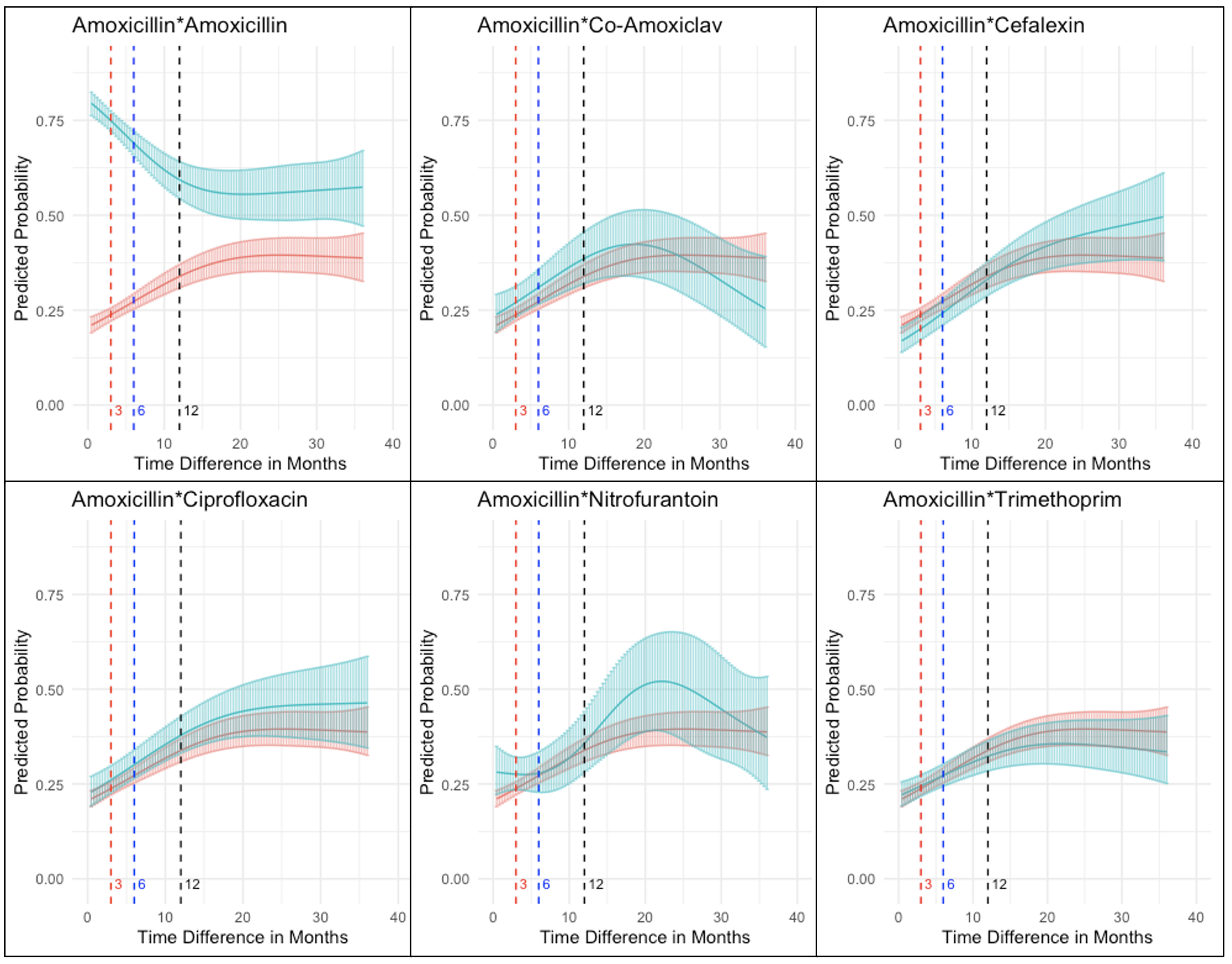


Note: Models control for AMR resistance to all antibiotics of interest at first infection other than pivmecillinam and fosfomycin (excluded as the numbers of resistant organisms and number of cultures reporting sensitivity results were low) and their interactions with continuous inter-infection time, calendar year at second infection, and age at second infection. For predictions, we assumed median age and the calendar year 2016 (as it represents the middle of the study period), and that the first infection was ‘susceptible’ to all other antibiotics. In panel labels “Amoxicillin*ciprofloxacin” reflects outcome*exposure; so the outcome being predicted is amoxicillin resistance in the second UTI, as a function of ciprofloxacin resistance in the first UTI (blue resistant, red susceptible), assuming the first UTI is resistant only to ciprofloxacin and not the other antibiotics shown. Red and blue curves overlapping indicates that there is no evidence that resistance to that antibiotic in the first UTI affects resistance to amoxicillin in the second UTI.

**Figure S5:** Predicted probability models of **co-amoxiclav** resistance at second infection, given resistance (blue) or susceptibility (red) to the indicated antibiotic at first infection, by inter-infection time, controlling for all antibiotic susceptibilities at first infection (multi-antibiotic models)


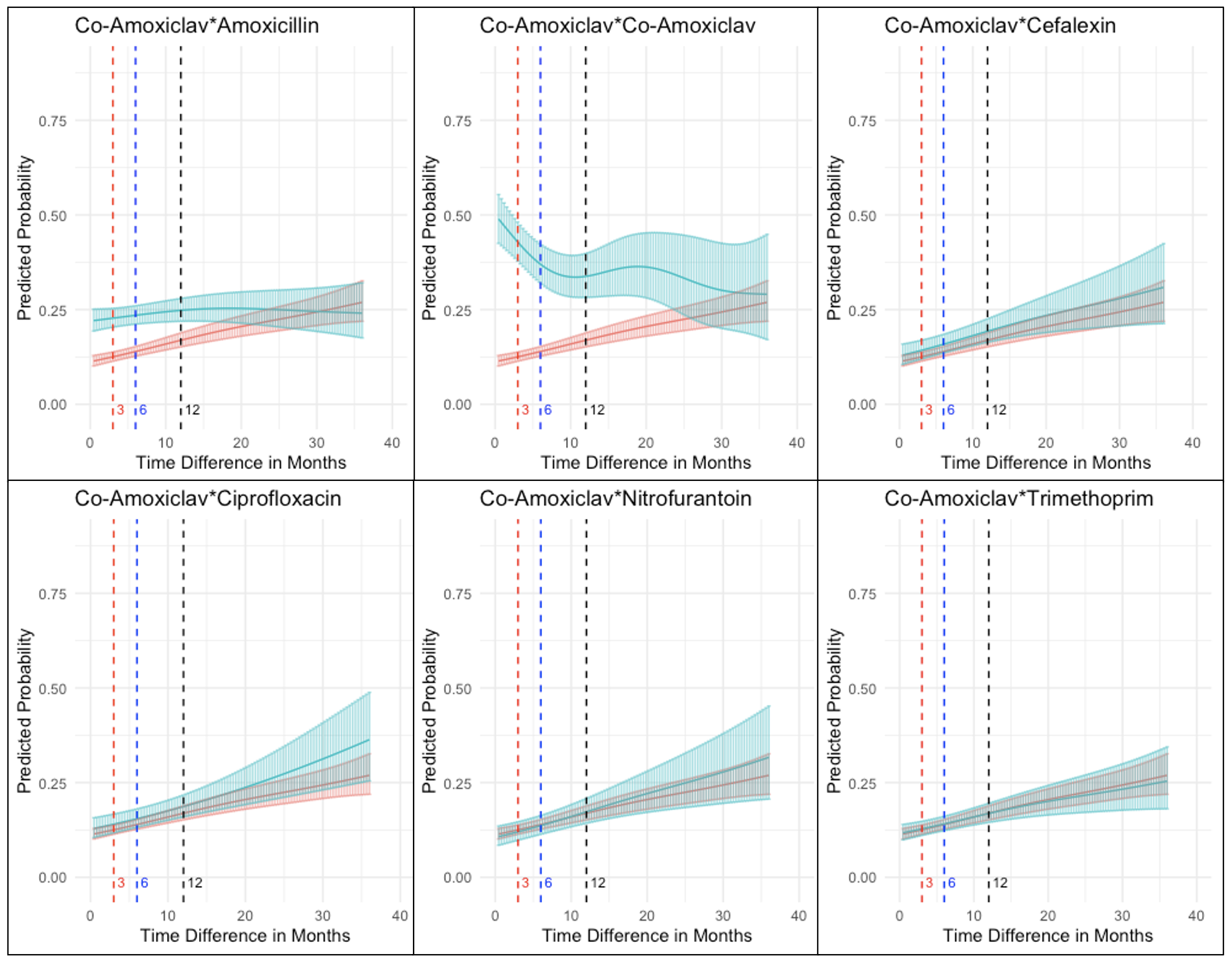


Note: Models control for AMR resistance to all antibiotics of interest at first infection other than pivmecillinam and fosfomycin (excluded as the numbers of resistant organisms and number of cultures reporting sensitivity results were low) and their interactions with continuous inter-infection time, calendar year at second infection, and age at second infection. For predictions, we assumed median age and the calendar year 2016 (as it represents the middle of the study period), and that the first infection was ‘susceptible’ to all other antibiotics. In panel labels “Co-amoxiclav*ciprofloxacin” reflects outcome*exposure; so the outcome being predicted is co-amoxiclav resistance in the second UTI, as a function of ciprofloxacin resistance in the first UTI (blue resistant, red susceptible), assuming the first UTI is resistant only to ciprofloxacin and not the other antibiotics shown. Red and blue curves overlapping indicates that there is no evidence that resistance to that antibiotic in the first UTI affects resistance to co-amoxiclav in the second UTI.

**Figure S6:** Predicted probability models of **cefalexin** resistance at second infection, given resistance (blue) or susceptibility (red) to the indicated antibiotic at first infection, by inter-infection time, controlling for all antibiotic susceptibilities at first infection (multi-antibiotic models)


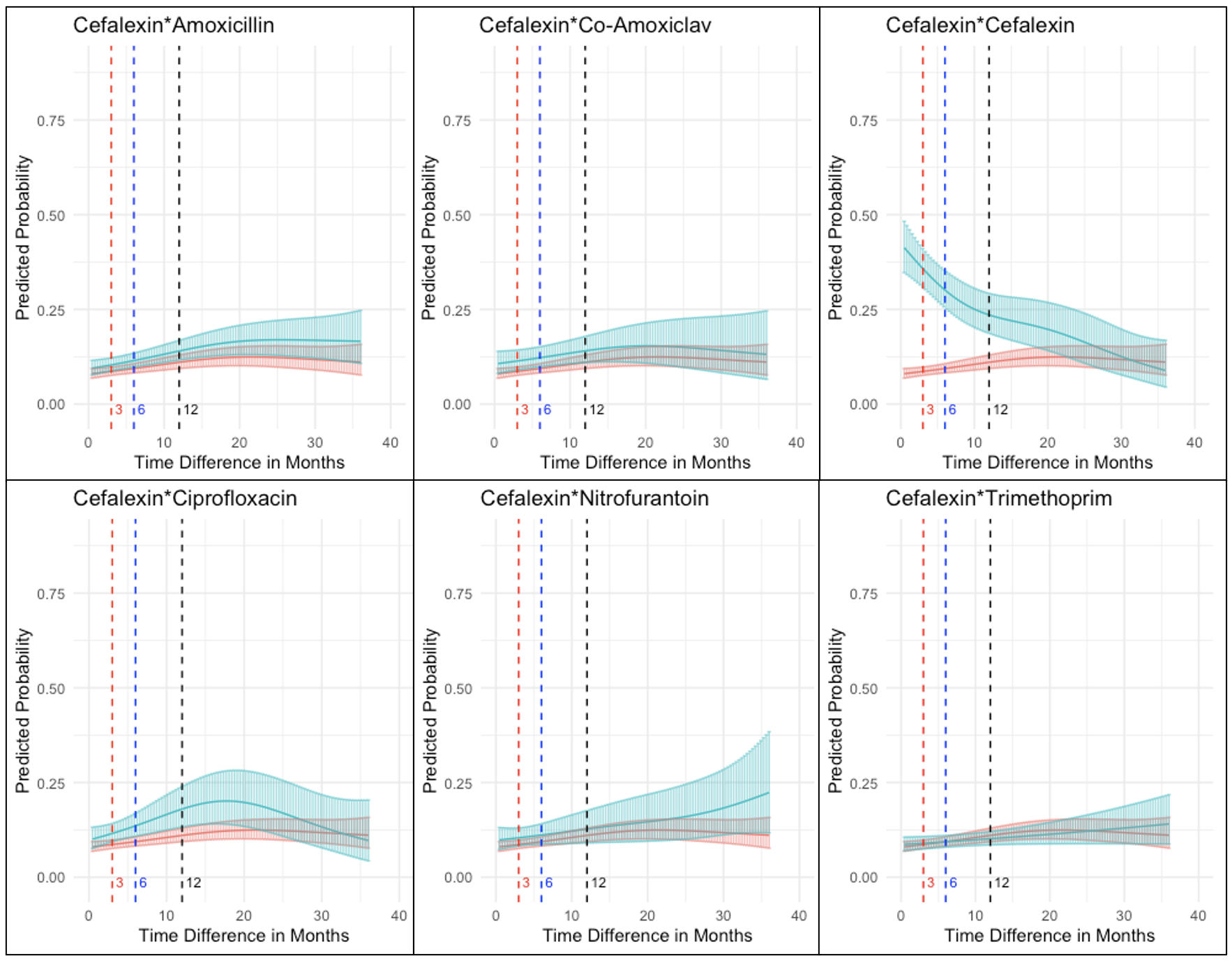


Note: Models control for AMR resistance to all antibiotics of interest at first infection other than pivmecillinam and fosfomycin (excluded as the numbers of resistant organisms and number of cultures reporting sensitivity results were low) and their interactions with continuous inter-infection time, calendar year at second infection, and age at second infection. For predictions, we assumed median age and the calendar year 2016 (as it represents the middle of the study period), and that the first infection was ‘susceptible’ to all other antibiotics. In panel labels “Cefalexin*ciprofloxacin” reflects outcome*exposure; so the outcome being predicted is cefalexin resistance in the second UTI, as a function of ciprofloxacin resistance in the first UTI (blue resistant, red susceptible), assuming the first UTI is resistant only to ciprofloxacin and not the other antibiotics shown. Red and blue curves overlapping indicates that there is no evidence that resistance to that antibiotic in the first UTI affects resistance to cefalexin in the second UTI.

**Figure S7:** Predicted probability models of **ciprofloxacin** resistance at second infection, given resistance (blue) or susceptibility (red) to the indicated antibiotic at first infection, by inter-infection time, controlling for all antibiotic susceptibilities at first infection (multi-antibiotic models)


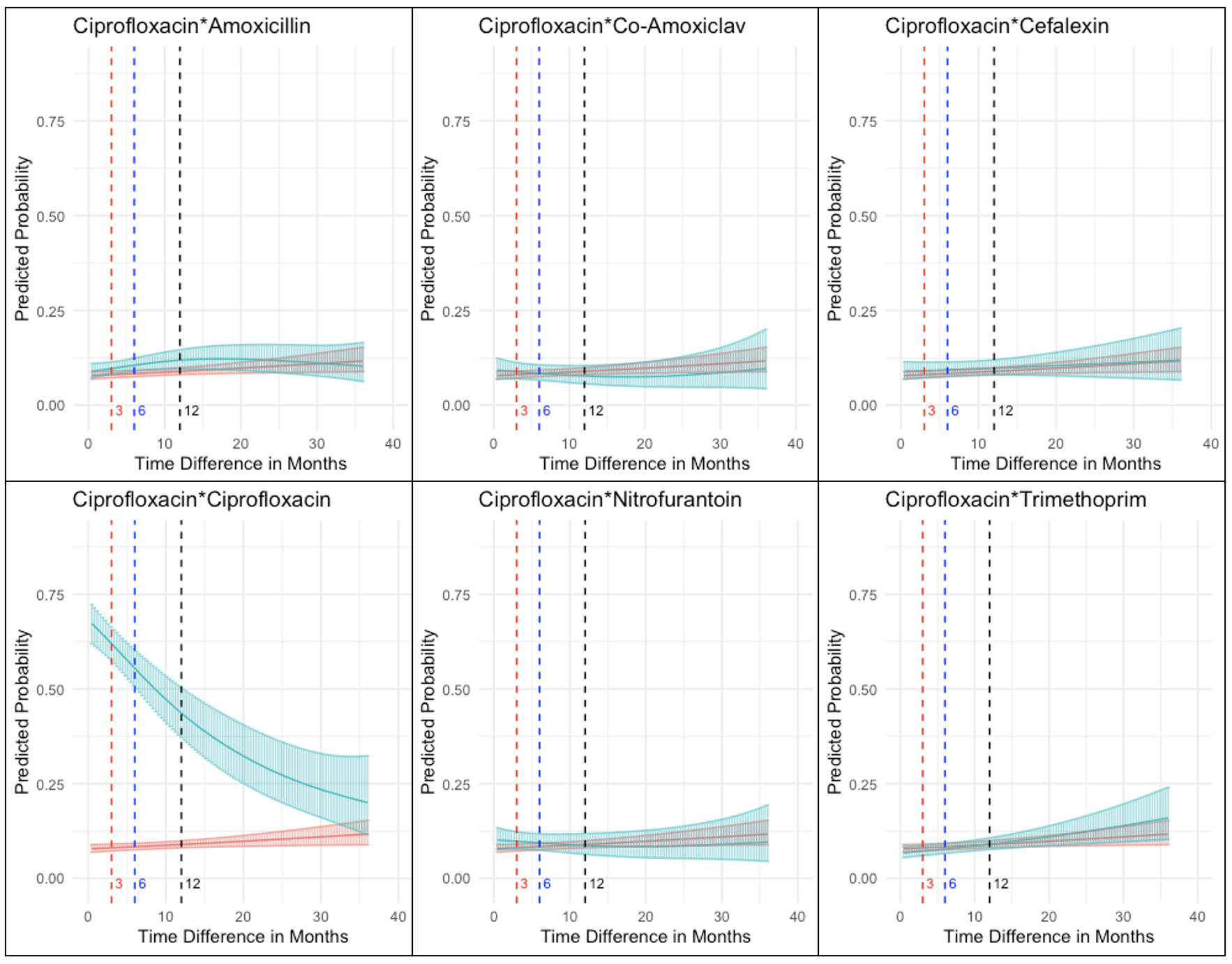


Note: Models control for AMR resistance to all antibiotics of interest at first infection other than pivmecillinam and fosfomycin (excluded as the numbers of resistant organisms and number of cultures reporting sensitivity results were low) and their interactions with continuous inter-infection time, calendar year at second infection, and age at second infection. For predictions, we assumed median age and the calendar year 2016 (as it represents the middle of the study period), and that the first infection was ‘susceptible’ to all other antibiotics. In panel labels “ciprofloxacin*amoxicillin” reflects outcome*exposure; so the outcome being predicted is ciprofloxacin resistance in the second UTI, as a function of amoxicillin resistance in the first UTI (blue resistant, red susceptible), assuming the first UTI is resistant only to amoxicillin and not the other antibiotics shown. Red and blue curves overlapping indicates that there is no evidence that resistance to that antibiotic in the first UTI affects resistance to ciprofloxacin in the second UTI.

**Figure S8:** Predicted probability models of **nitrofurantoin** resistance at second infection, given resistance (blue) or susceptibility (red) to the indicated antibiotic at first infection, by inter-infection time, controlling for all antibiotic susceptibilities at first infection (multi-antibiotic models)


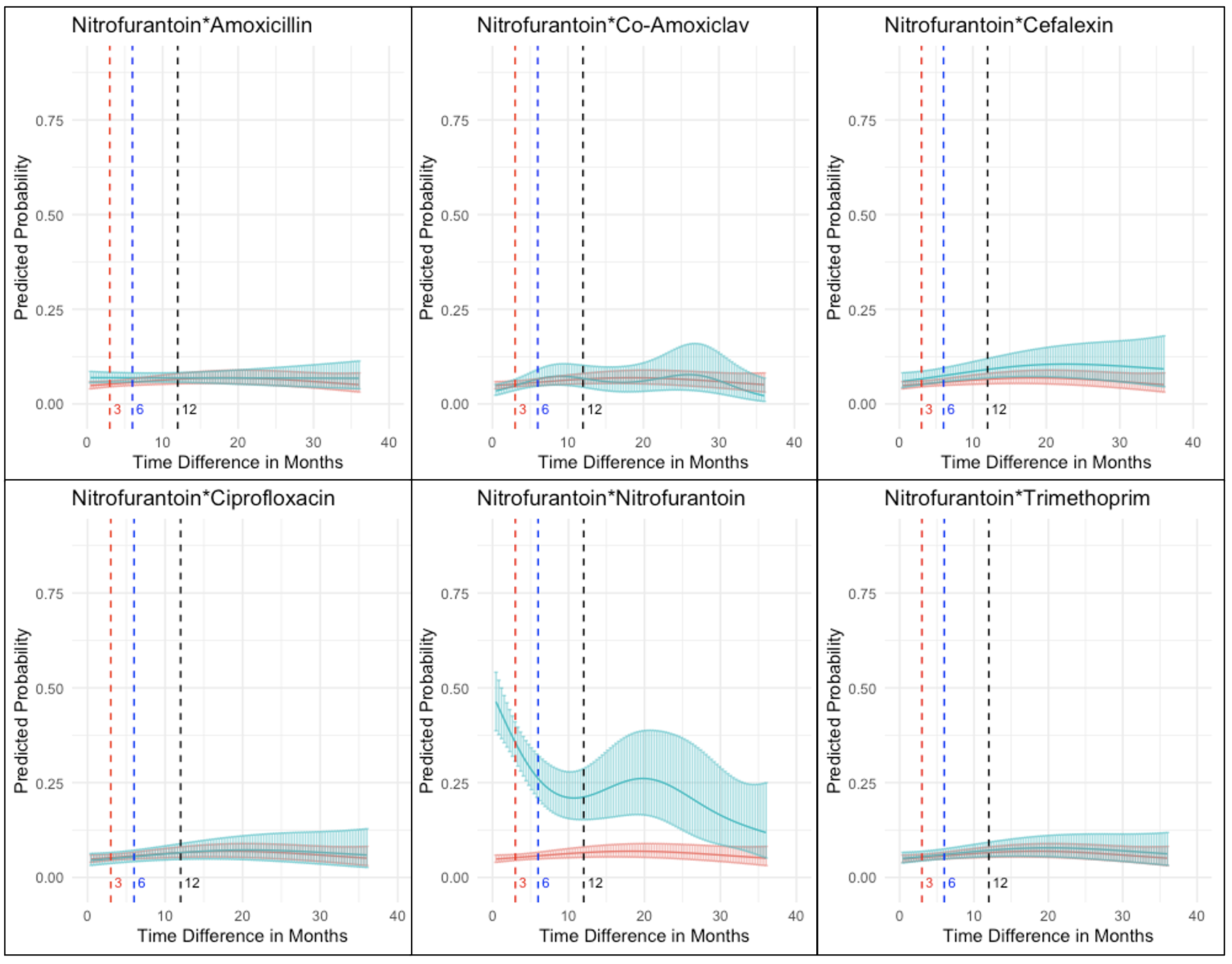


Note: Models control for AMR resistance to all antibiotics of interest at first infection other than pivmecillinam and fosfomycin (excluded as the numbers of resistant organisms and number of cultures reporting sensitivity results were low) and their interactions with continuous inter-infection time, calendar year at second infection, and age at second infection. For predictions, we assumed median age and the calendar year 2016 (as it represents the middle of the study period), and that the first infection was ‘susceptible’ to all other antibiotics. In panel labels “nitrofurantoin*ciprofloxacin” reflects outcome*exposure; so the outcome being predicted is nitrofurantoin resistance in the second UTI, as a function of ciprofloxacin resistance in the first UTI (blue resistant, red susceptible), assuming the first UTI is resistant only to ciprofloxacin and not the other antibiotics shown. Red and blue curves overlapping indicates that there is no evidence that resistance to that antibiotic in the first UTI affects resistance to nitrofurantoin in the second UTI.

**Figure S9:** Predicted probability models of **trimethoprim** resistance at second infection, given resistance (blue) or susceptibility (red) to the indicated antibiotic at first infection, by inter-infection time, controlling for all antibiotic susceptibilities at first infection (multi-antibiotic models)


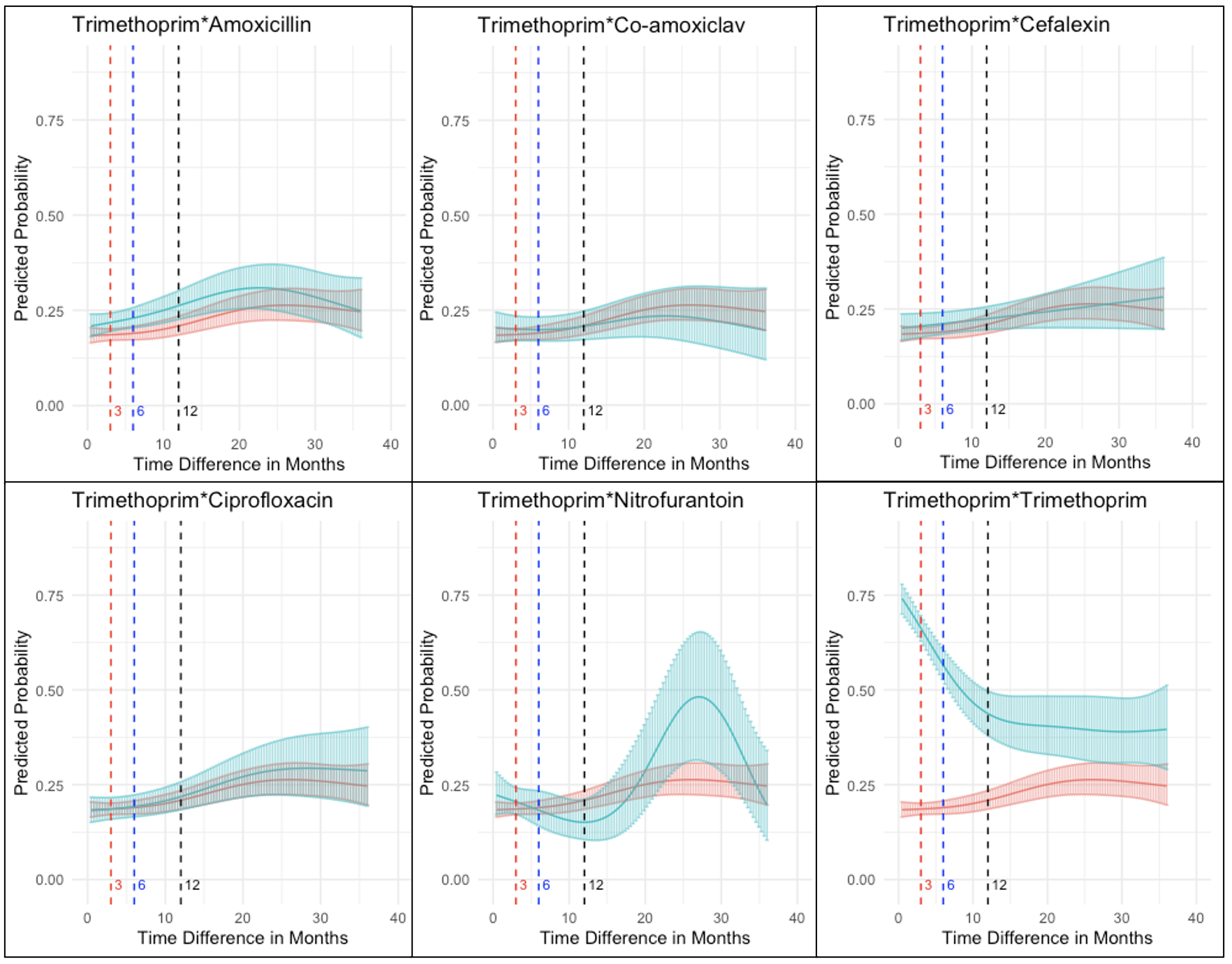


Note: Models control for AMR resistance to all antibiotics of interest at first infection other than pivmecillinam and fosfomycin (excluded as the numbers of resistant organisms and number of cultures reporting sensitivity results were low) and their interactions with continuous inter-infection time, calendar year at second infection, and age at second infection. For predictions, we assumed median age and the calendar year 2016 (as it represents the middle of the study period), and that the first infection was ‘susceptible’ to all other antibiotics. In panel labels “trimethoprim*ciprofloxacin” reflects outcome*exposure; so the outcome being predicted is trimethoprim resistance in the second UTI, as a function of ciprofloxacin resistance in the first UTI (blue resistant, red susceptible), assuming the first UTI is resistant only to ciprofloxacin and not the other antibiotics shown. Red and blue curves overlapping indicates that there is no evidence that resistance to that antibiotic in the first UTI affects resistance to trimethoprim in the second UTI.

**Figure S10:** Predicted probability models of **amoxicillin** resistance at second infection, given resistance (blue) or susceptibility (red) to the indicated antibiotic at first infection, by inter-infection time, controlling for all antibiotic susceptibilities at first infection (multi-antibiotic models) **in UTI pairs caused by *E. coli.***


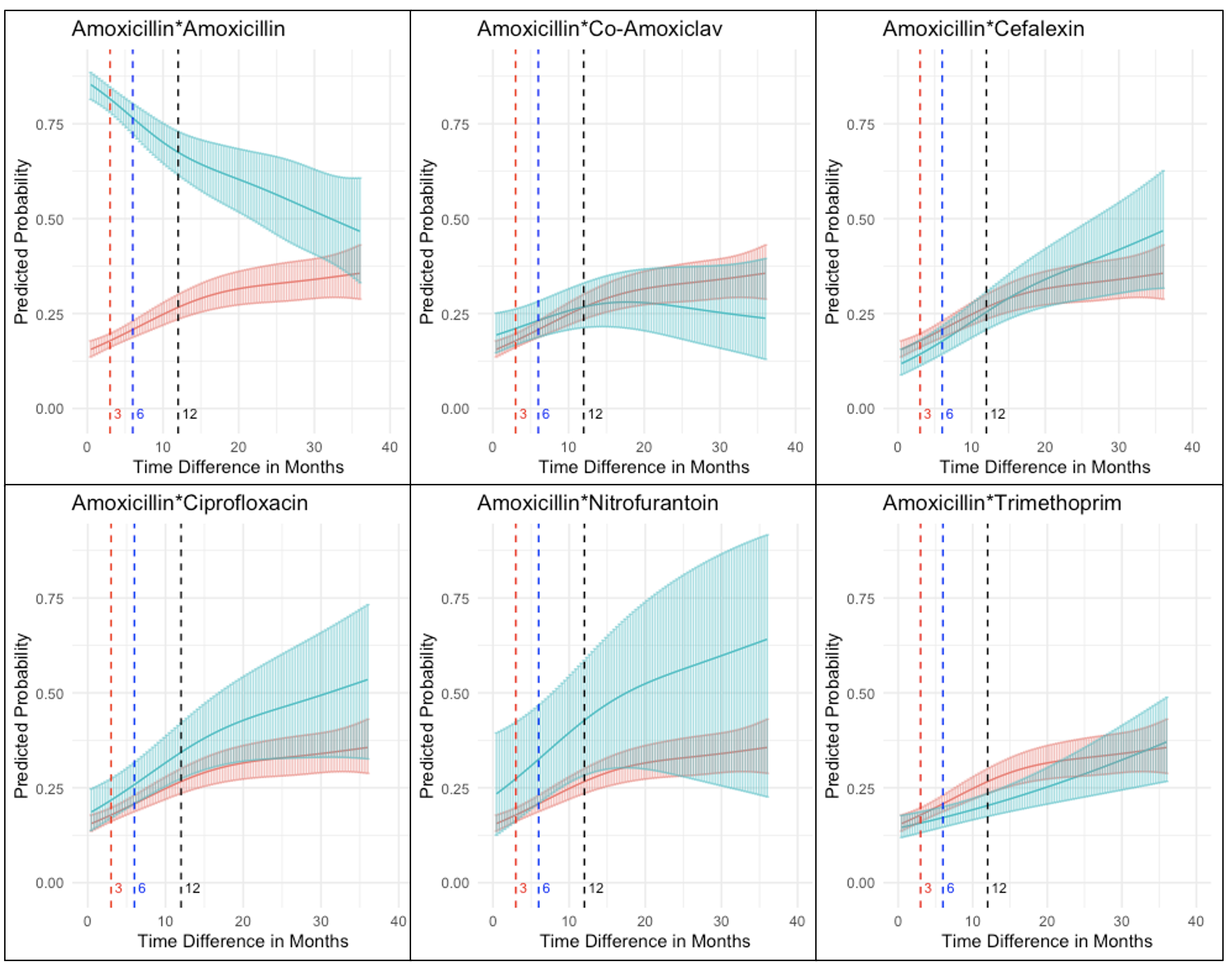


Note: Models control for AMR resistance to all antibiotics of interest at first infection other than pivmecillinam and fosfomycin (excluded as the numbers of resistant organisms and number of cultures reporting sensitivity results were low) and their interactions with continuous inter-infection time, calendar year at second infection, and age at second infection. For predictions, we assumed median age and the calendar year 2016 (as it represents the middle of the study period), and that the first infection was ‘susceptible’ to all other antibiotics. In panel labels “amoxicillin*ciprofloxacin” reflects outcome*exposure; so the outcome being predicted is amoxicillin resistance in the second UTI, as a function of ciprofloxacin resistance in the first UTI (blue resistant, red susceptible), assuming the first UTI is resistant only to ciprofloxacin and not the other antibiotics shown. Red and blue curves overlapping indicates that there is no evidence that resistance to that antibiotic in the first UTI affects resistance to amoxicillin in the second UTI in UTI pairs caused by *E. coli*.

**Figure S11:** Predicted probability models of **co-amoxiclav** resistance at second infection, given resistance (blue) or susceptibility (red) to the indicated antibiotic at first infection, by inter-infection time, controlling for all antibiotic susceptibilities at first infection (multi-antibiotic models) **in UTI pairs caused by *E. coli.***


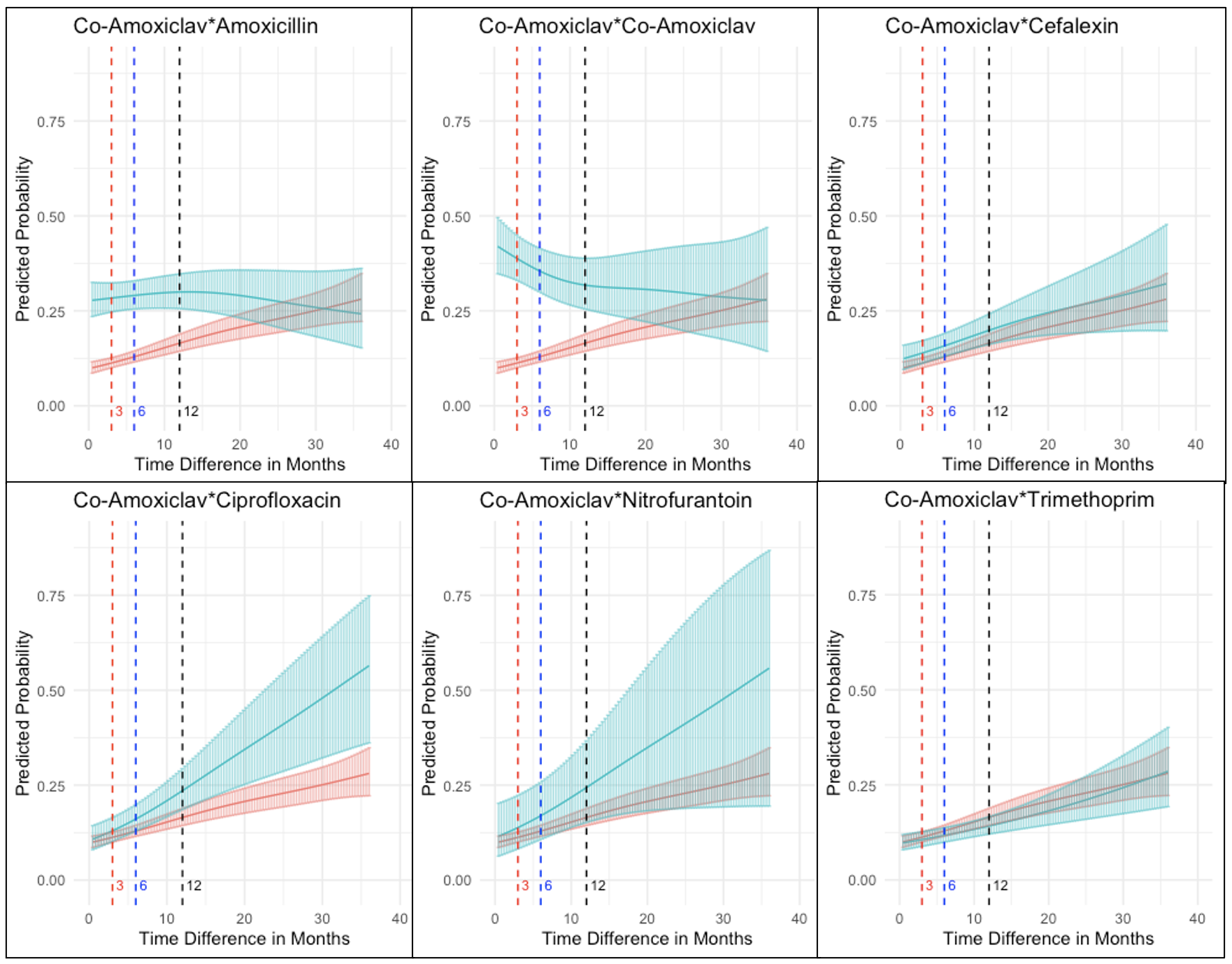


Note: Models control for AMR resistance to all antibiotics of interest at first infection other than pivmecillinam and fosfomycin (excluded as the numbers of resistant organisms and number of cultures reporting sensitivity results were low) and their interactions with continuous inter-infection time, calendar year at second infection, and age at second infection. For predictions, we assumed median age and the calendar year 2016 (as it represents the middle of the study period), and that the first infection was ‘susceptible’ to all other antibiotics. In panel labels “co-amoxiclav*ciprofloxacin” reflects outcome*exposure; so the outcome being predicted is co-amoxiclav resistance in the second UTI, as a function of ciprofloxacin resistance in the first UTI (blue resistant, red susceptible), assuming the first UTI is resistant only to ciprofloxacin and not the other antibiotics shown. Red and blue curves overlapping indicates that there is no evidence that resistance to that antibiotic in the first UTI affects resistance to co-amoxiclav in the second UTI in UTI pairs caused by *E. coli*.

**Figure S12:** Predicted probability models of **cefalexin** resistance at second infection, given resistance (blue) or susceptibility (red) to the indicated antibiotic at first infection, by inter-infection time, controlling for all antibiotic susceptibilities at first infection (multi-antibiotic models) **in UTI pairs caused by *E. coli.***


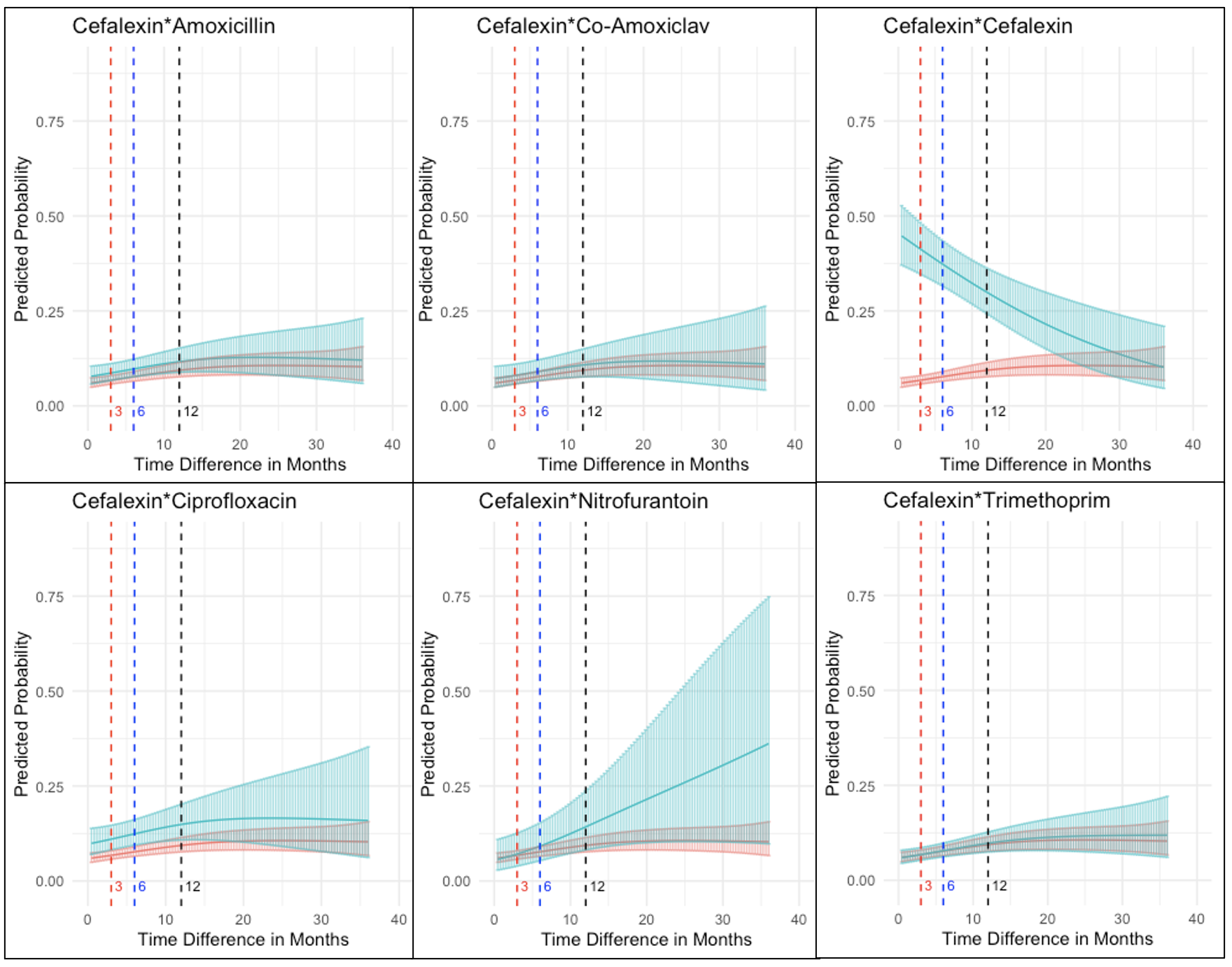


Note: Models control for AMR resistance to all antibiotics of interest at first infection other than pivmecillinam and fosfomycin (excluded as the numbers of resistant organisms and number of cultures reporting sensitivity results were low) and their interactions with continuous inter-infection time, calendar year at second infection, and age at second infection. For predictions, we assumed median age and the calendar year 2016 (as it represents the middle of the study period), and that the first infection was ‘susceptible’ to all other antibiotics. In panel labels “cefalexin*ciprofloxacin” reflects outcome*exposure; so the outcome being predicted is cefalexin resistance in the second UTI, as a function of ciprofloxacin resistance in the first UTI (blue resistant, red susceptible), assuming the first UTI is resistant only to ciprofloxacin and not the other antibiotics shown. Red and blue curves overlapping indicates that there is no evidence that resistance to that antibiotic in the first UTI affects resistance to cefalexin in the second UTI in UTI pairs caused by *E. coli*.

**Figure S13:** Predicted probability models of **ciprofloxacin** resistance at second infection, given resistance (blue) or susceptibility (red) to the indicated antibiotic at first infection, by inter-infection time, controlling for all antibiotic susceptibilities at first infection (multi-antibiotic models) **in UTI pairs caused by *E. coli.***


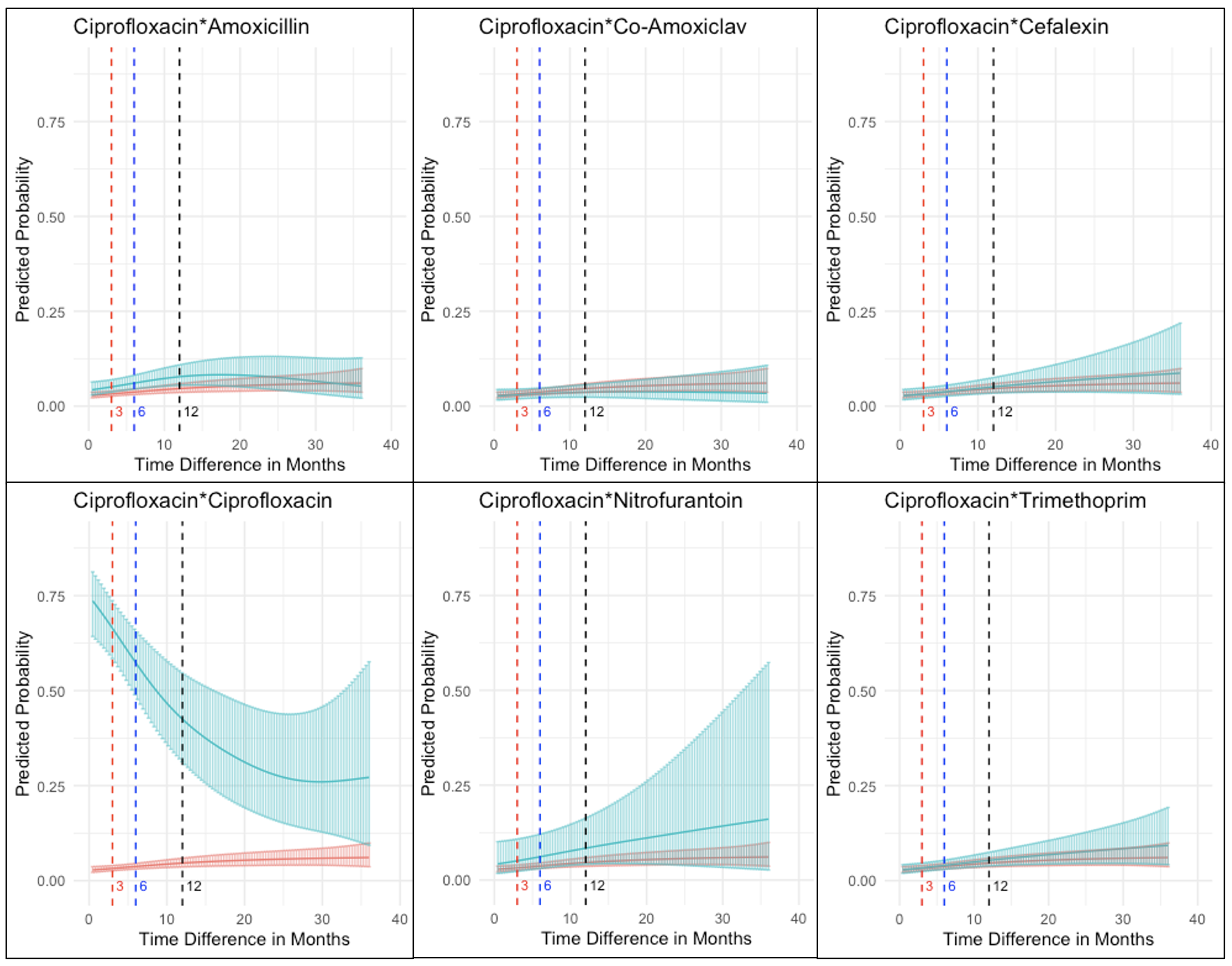


Note: Models control for AMR resistance to all antibiotics of interest at first infection other than pivmecillinam and fosfomycin (excluded as the numbers of resistant organisms and number of cultures reporting sensitivity results were low) and their interactions with continuous inter-infection time, calendar year at second infection, and age at second infection. For predictions, we assumed median age and the calendar year 2016 (as it represents the middle of the study period), and that the first infection was ‘susceptible’ to all other antibiotics. In panel labels “ciprofloxacin*cefalexin” reflects outcome*exposure; so the outcome being predicted is ciprofloxacin resistance in the second UTI, as a function of cefalexin resistance in the first UTI (blue resistant, red susceptible), assuming the first UTI is resistant only to cefalexin and not the other antibiotics shown. Red and blue curves overlapping indicates that there is no evidence that resistance to that antibiotic in the first UTI affects resistance to ciprofloxacin in the second UTI in UTI pairs caused by *E. coli*.

**Figure S14:** Predicted probability models of **nitrofurantoin** resistance at second infection, given resistance (blue) or susceptibility (red) to the indicated antibiotic at first infection, by inter-infection time, controlling for all antibiotic susceptibilities at first infection (multi-antibiotic models) **in UTI pairs caused by *E. coli.***


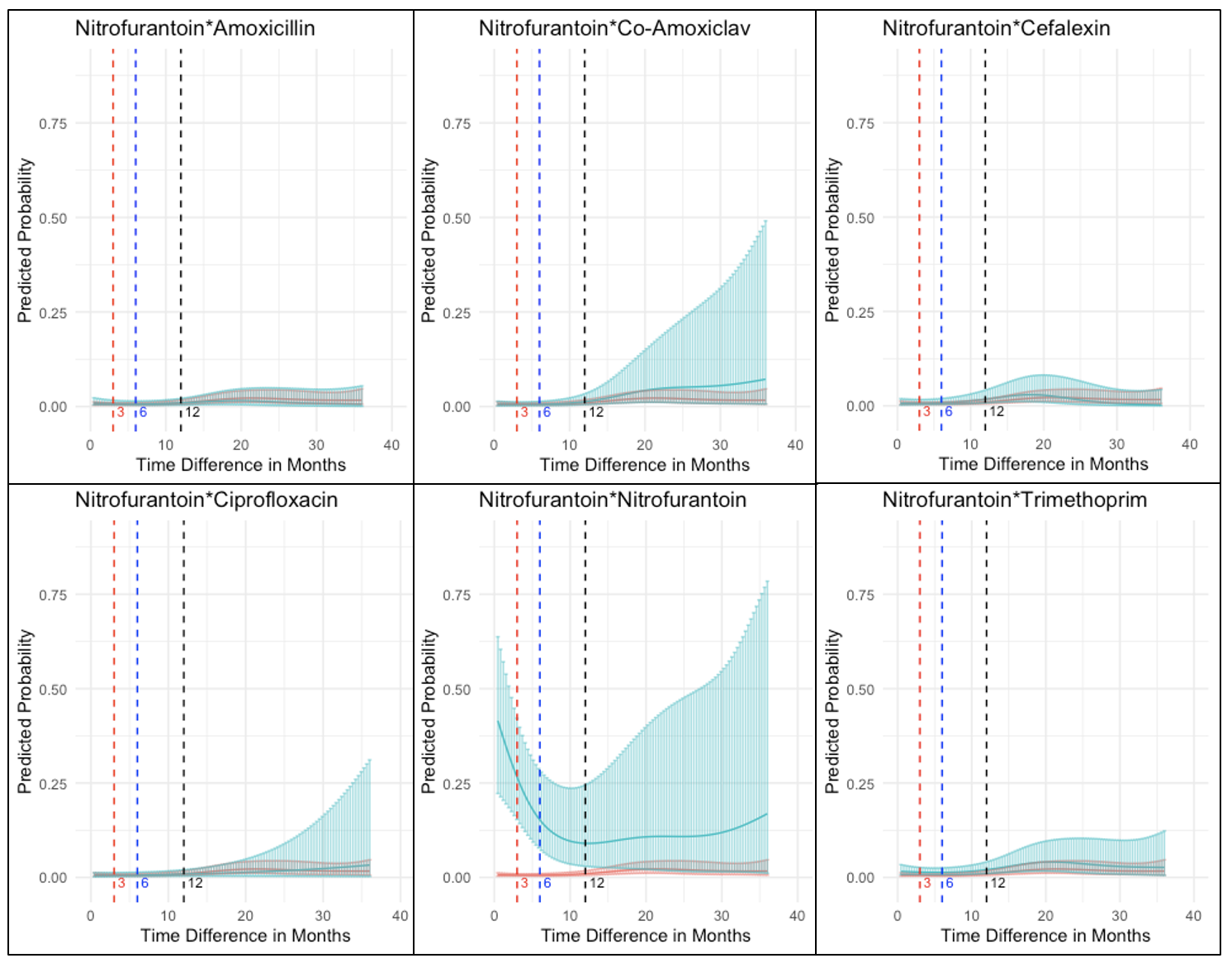


Note: Models control for AMR resistance to all antibiotics of interest at first infection other than pivmecillinam and fosfomycin (excluded as the numbers of resistant organisms and number of cultures reporting sensitivity results were low) and their interactions with continuous inter-infection time, calendar year at second infection, and age at second infection. For predictions, we assumed median age and the calendar year 2016 (as it represents the middle of the study period), and that the first infection was ‘susceptible’ to all other antibiotics. In panel labels “nitrofurantoin*ciprofloxacin” reflects outcome*exposure; so the outcome being predicted is nitrofurantoin resistance in the second UTI, as a function of ciprofloxacin resistance in the first UTI (blue resistant, red susceptible), assuming the first UTI is resistant only to ciprofloxacin and not the other antibiotics shown. Red and blue curves overlapping indicates that there is no evidence that resistance to that antibiotic in the first UTI affects resistance to nitrofurantoin in the second UTI in UTI pairs caused by *E. coli*.

**Figure S15:** Predicted probability models of **trimethoprim** resistance at second infection, given resistance (blue) or susceptibility (red) to the indicated antibiotic at first infection, by inter-infection time, controlling for all antibiotic susceptibilities at first infection (multi-antibiotic models) **in UTI pairs caused by *E. coli.***


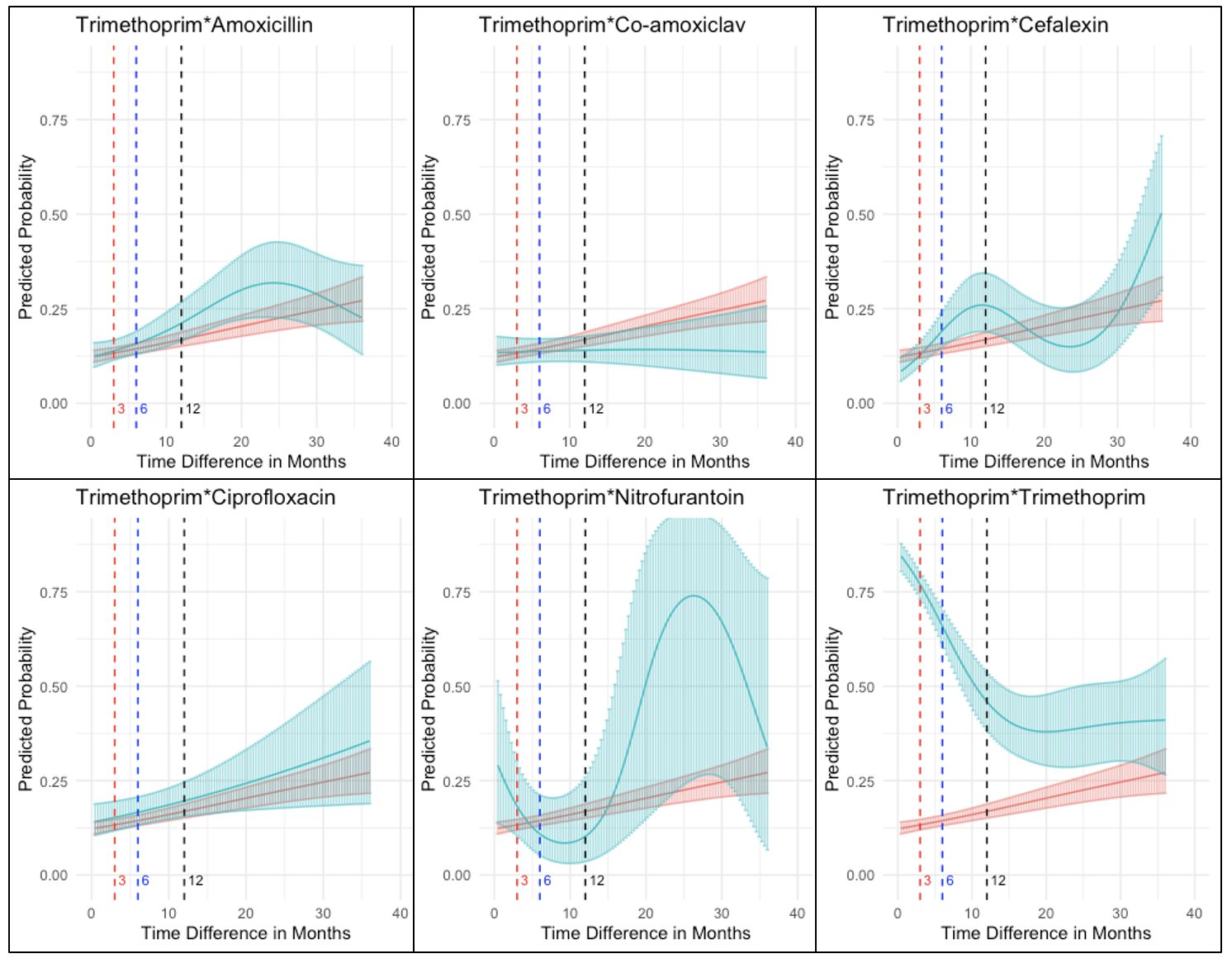


Note: Models control for AMR resistance to all antibiotics of interest at first infection other than pivmecillinam and fosfomycin (excluded as the numbers of resistant organisms and number of cultures reporting sensitivity results were low) and their interactions with continuous inter-infection time, calendar year at second infection, and age at second infection. For predictions, we assumed median age and the calendar year 2016 (as it represents the middle of the study period), and that the first infection was ‘susceptible’ to all other antibiotics. In panel labels “trimethoprim*ciprofloxacin” reflects outcome*exposure; so the outcome being predicted is trimethoprim resistance in the second UTI, as a function of ciprofloxacin resistance in the first UTI (blue resistant, red susceptible), assuming the first UTI is resistant only to ciprofloxacin and not the other antibiotics shown. Red and blue curves overlapping indicates that there is no evidence that resistance to that antibiotic in the first UTI affects resistance to trimethoprim in the second UTI in UTI pairs caused by *E. coli*.

**Table S1:** Association between age at second infection and AMR resistance at second infection in single and multi-antibiotic models.

|  | Single antibiotic  All species | Single antibiotic  *e. coli* | multi-antibiotic  all species | multi-antibiotic  *e. coli* |
| --- | --- | --- | --- | --- |
| Amoxicillin | **1.07**  **(1.05, 1.10)**  **P<0.001** | 1.03  (1.00, 1.06)  P=0.050 | **1.06**  **(1.04, 1.09)**  **P<0.001** | 1.03  (0.99, 1.06)  P=0.158 |
| Co-Amoxiclav | **1.05**  **(1.03, 1.08)**  **P<0.001** | **1.05**  **(1.02, 1.09)**  **P<0.001** | **1.05**  **(1.02, 1.08)**  **P<0.001** | **1.06**  **(1.03, 1.09)**  **P=0.001** |
| Cefalexin | **1.07**  **(1.04, 1.11)**  **P<0.001** | **1.06**  **(1.02, 1.11)**  **P=0.004** | **1.07**  **(1.04, 1.11)**  **P<0.001** | **1.06**  **(1.02, 1.11)**  **P=0.004** |
| Ciprofloxacin | 0.98  (0.95, 1.01)  P=0.200 | 1.04  (0.99, 1.10)  P=0.166 | 0.99  (0.96, 1.02)  P=0.386 | 1.03  (0.98, 1.09)  P=0.244 |
| Fosfomycin | **1.06**  **(1.00, 1.12)**  **P=0.044** | **1.16**  **(1.01, 1.35)**  **P=0.040** | ** | ** |
| Nitrofurantoin | **1.14**  **(1.10, 1.19)**  **P<0.001** | **1.26**  **(1.12, 1.43)**  **P<0.001** | **1.13**  **(1.08, 1.17)**  **P<0.001** | **1.27**  **(1.12, 1.46)**  **P<0.001** |
| Pivmecillinam | 1.02  (0.95, 1.09)  P=0.636 | 1.02  (0.95, 1.10)  P=0.545 | ** | ** |
| Trimethoprim | 1.00  (0.97, 1.03)  P=0.920 | 1.00  (0.97, 1.04)  P=0.782 | 1.00  (0.98, 1.03)  P=0.920 | 1.00  (0.97, 1.04)  P=0.882 |

Note: Single antibiotic models control for AMR resistance to the same antibiotic at first infection and its interactions with inter-infection time, and calendar year at second infection. Multi-antibiotic models control for AMR resistance to all antibiotics at first infection and their interactions with inter-infection time, and calendar year at second infection. OR is odds ratio per 10 years older. CI is confidence interval.

** Pivmecillinam and fosfomycin were excluded from multi-antibiotic models as the numbers of resistant organisms and number of cultures reporting sensitivity results were low.

**Table S2:** Association between calendar year at second infection and AMR resistance at second infection in single and multi-antibiotic models.

|  | Single antibiotic  All species | Single antibiotic  *e. coli* | multi-antibiotic  all species | multi-antibiotic  *e. coli* |
| --- | --- | --- | --- | --- |
| Amoxicillin | **0.96**  **(0.94, 0.99)**  **P=0.007** | 0.98  (0.94, 1.02)  P=0.249 | **0.96**  **(0.93, 0.99)**  **P=0.009** | 0.97  (0.93, 1.01)  P=0.109 |
| Co-Amoxiclav | 0.99  (0.96, 1.03)  P=0.634 | 1.02  (0.98, 1.06)  P=0.339 | 1.00  (0.97, 1.04)  P=0.870 | 1.04  (1.00, 1.08)  P=0.084 |
| Cefalexin | **0.80**  **(0.77, 0.83)**  **P<0.001** | **0.82**  **0.78, 0.86)**  **P<0.001** | **0.80**  **(0.77, 0.83)**  **P<0.001** | **0.81**  **(0.77, 0.86)**  **P<0.001** |
| Ciprofloxacin | **1.07**  **(1.03, 1.11)**  **P<0.001** | 1.07  (1.00, 1.14)  P=0.057 | **1.06**  **(1.02, 1.11)**  **P=0.003** | 1.07  (1.00, 1.15)  P=0.065 |
| Fosfomycin | 0.97  (0.90, 1.04)  P=0.381 | **0.84**  **(0.71, 0.99)**  **P=0.043** | ** | ** |
| Nitrofurantoin | 0.98  (0.93, 1.02)  P=0.316 | 0.97  (0.85, 1.11)  P=0.655 | 0.99  (0.94, 1.04)  P=0.739 | 1.01  (0.88, 1.16)  P=0.906 |
| Pivmecillinam | 0.97  (0.84, 1.12)  P=0.691 | 0.99  (0.85, 1.14)  P=0.871 | ** | ** |
| Trimethoprim | 0.99  (0.96, 1.02)  P=0.693 | **0.96**  **(0.92, 1.00)**  **P=0.04** | 0.99  (0.96, 1.02)  P=0.563 | **0.95**  **(0.91, 1.00)**  **P=0.031** |

Note: Single antibiotic models control for AMR resistance to the same antibiotic at first infection and its interactions with inter-infection time, and age. Multi-antibiotic models control for AMR resistance to all antibiotic at first infection and their interactions with inter-infection time, and calendar year at second infection. OR is odds ratio per one year later. CI is confidence interval.

** Pivmecillinam and fosfomycin were excluded from multi-antibiotic models as the numbers of resistant organisms and number of cultures reporting sensitivity results were low.

**Table S3:** Associations between **amoxicillin** resistance at second infection with resistance to all antibiotic of interest at first infection, by inter-infection time, controlling for all antibiotic susceptibilities at first infection (multi-antibiotic models).

|  | 0 to <3  months  OR (95% CI) | 3 to <6  months  OR (95% CI) | 6 to <12 months  OR (95% CI) | ≥12  Months  OR (95% CI) |
| --- | --- | --- | --- | --- |
| Amoxicillin | **13.03**  **(10.67, 15.92)**  **P< 0.001** | **6.10**  **(4.63, 8.04)**  **P<0.001** | **4.27**  **(3.04, 6.01)**  **P<0.001** | **2.04**  **(1.58, 2.63)**  **P<0.001** |
| Co-Amoxiclav | 1.09  (0.85, 1.40)  P=0.490 | 1.23  (0.89, 1.71)  P=0.208 | 1.45  (0.93, 2.25)  P=0.098 | 0.97  (0.71, 1.34)  P=0.869 |
| Cefalexin | **0.78**  **(0.62, 0.99)**  **P=0.041** | 0.85  (0.62, 1.17)  P=0.327 | 0.72  (0.48, 1.08)  P=0.108 | 1.28  (0.97, 1.69)  P=0.081 |
| Ciprofloxacin | 1.16  (0.91, 1.46)  P=0.227 | 1.05  (0.74, 1.48)  P=0.789 | 1.07  (0.68, 1.68)  P=0.784 | 1.36  (0.98, 1.88)  P=0.064 |
| Nitrofurantoin | **1.40**  **(1.07, 1.83)**  **P=0.014** | 0.91  (0.62, 1.35)  P=0.654 | 1.31  (0.82, 2.07)  P=0.257 | 1.19  (0.83, 1.71)  P=0.334 |
| Trimethoprim | 1.07  (0.89, 1.28)  P=0.497 | 1.02  (0.79, 1.31)  P=0.880 | 1.07  (0.77, 1.49)  P=0.697 | **0.78**  **(0.62, 1.00)**  **P= 0.045** |

Note: Models control for AMR resistance to all antibiotics shown at the first infection and their interactions with inter-infection time, calendar year at second infection, and age at second infection. OR is odds ratio. CI is confidence interval. Pivmecillinam and fosfomycin were excluded from multi-antibiotic models as the numbers of resistant organisms and number of cultures reporting sensitivity results were low.

**Table S4:** Associations between **co-amoxiclav** resistance at second infection with resistance to all antibiotic of interest at first infection, by inter-infection time, controlling for all antibiotic susceptibilities at first infection (multi-antibiotic models).

|  | 0 to <3  months  OR (95% CI) | 3 to <6  months  OR (95% CI) | 6 to <12 months  OR (95% CI) | ≥12  Months  OR (95% CI) |
| --- | --- | --- | --- | --- |
| Amoxicillin | **2.08**  **(1.67, 2.59)**  **P<0.001** | **1.86**  **(1.33, 2.60)**  **P<0.001** | **2.34**  **(1.57, 3.50)**  **P<0.001** | 1.07  (0.79, 1.44)  P=0.677 |
| Co-Amoxiclav | **6.38**  **(5.07, 8.03)**  **P<0.001** | **4.27**  **(3.03, 6.02)**  **P<0.001** | **2.93**  **(1.87, 4.59)**  **P<0.001** | **1.91**  **(1.34, 2.72)**  **P<0.001** |
| Cefalexin | 1.14  (0.90, 1.43)  P=0.275 | 1.35  (0.98, 1.87)  P=0.067 | 0.91  (0.60, 1.38)  P=0.652 | 1.16  (0.86, 1.58)  P=0.702 |
| Ciprofloxacin | 1.15  (0.90, 1.47)  P=0.277 | 0.94  (0.64, 1.38)  P=0.762 | 1.36  (0.83, 2.23)  P=0.229 | 1.37  (0.96, 1.95)  P=0.085 |
| Nitrofurantoin | 0.98  (0.74, 1.29)  P=0.861 | 0.84  (0.53, 1.34)  P=0.460 | 1.16  (0.71, 1.92)  P=0.551 | 1.14  (0.77, 1.69)  P=0.515 |
| Trimethoprim | 1.06  (0.88, 1.28)  P=0.555 | 1.09  (0.83, 1.43)  P=0.553 | 0.85  (0.60, 1.22)  P=0.390 | 0.91  (0.70, 1.20)  P=0.508 |

Note: Models control for AMR resistance to all antibiotics shown at the first infection and their interactions with inter-infection time, calendar year at second infection, and age at second infection. OR is odds ratio. CI is confidence interval. Pivmecillinam and fosfomycin were excluded from multi-antibiotic models as the numbers of resistant organisms and number of cultures reporting sensitivity results were low.

**Table S5:** Associations between **cefalexin** resistance at second infection with resistance to all antibiotic of interest at first infection, by inter-infection time, controlling for all antibiotic susceptibilities at first infection (multi-antibiotic models).

|  | 0 to <3  months  OR (95% CI) | 3 to <6  months  OR (95% CI) | 6 to <12 months  OR (95% CI) | ≥12  Months  OR (95% CI) |
| --- | --- | --- | --- | --- |
| Amoxicillin | 1.15  (0.88, 1.49)  P=0.305 | 1.06  (0.71, 1.59)  P=0.765 | **2.37**  **(1.45, 3.88)**  **P=0.001** | 1.33  (0.93, 1.92)  P=0.121 |
| Co-Amoxiclav | **1.38**  **(1.04, 1.83)**  **P=0.026** | 1.37  (0.90, 2.10)  P=0.145 | 1.33  (0.78, 2.27)  P=0.299 | 1.29  (0.85, 1.98)  P=0.234 |
| Cefalexin | **7.05**  **(5.55, 8.95)**  **P<0.001** | **5.52**  **(3.84, 7.93)**  **P<0.001** | **2.35**  **(1.48, 3.74)**  **P<0.001** | **1.48**  **(1.03, 2.13)**  **P=0.035** |
| Ciprofloxacin | **1.32**  **(1.01, 1.73)**  **P=0.046** | 1.01  (0.66, 1.55)  P=0.957 | **2.51**  **(1.44, 4.35)**  **P=0.001** | **1.60**  **(1.06, 2.41)**  **P=0.025** |
| Nitrofurantoin | **1.35**  **(1.02, 1.80)**  **P=0.040** | 0.79  (0.47, 1.33)  P=0.370 | **1.89**  **(1.09, 3.29)**  **P=0.024** | 1.30  (0.84, 2.02)  P=0.247 |
| Trimethoprim | 1.11  (0.90, 1.38)  P=0.334 | 1.15  (0.83, 1.59)  P=0.406 | 0.71  (0.46, 1.09)  P=0.116 | 0.93  (0.67, 1.29)  P=0.653 |

Note: Models control for AMR resistance to all antibiotics shown at the first infection and their interactions with inter-infection time, calendar year at second infection, and age at second infection. OR is odds ratio. CI is confidence interval. Pivmecillinam and fosfomycin were excluded from multi-antibiotic models as the numbers of resistant organisms and number of cultures reporting sensitivity results were low.

**Table S6:** Associations between **ciprofloxacin** resistance at second infection with resistance to all antibiotic of interest at first infection, by inter-infection time, controlling for all antibiotic susceptibilities at first infection (multi-antibiotic models).

|  | 0 to <3  months  OR (95% CI) | 3 to <6  months  OR (95% CI) | 6 to <12 months  OR (95% CI) | ≥12  Months  OR (95% CI) |
| --- | --- | --- | --- | --- |
| Amoxicillin | 1.16  (0.89, 1.52)  P=0.280 | 1.10  (0.75, 1.62)  P=0.621 | **1.64**  **(1.01, 2.65)**  **P=0.044** | 1.22  (0.84, 1.76)  P=0.296 |
| Co-Amoxiclav | 1.25  (0.91, 1.70)  P=0.164 | 0.94  (0.60, 1.47)  P=0.787 | 1.08  (0.61, 1.93)  P=0.785 | 0.71  (0.44, 1.14)  P=0.154 |
| Cefalexin | 1.12  (0.84, 1.49)  P=0.451 | 1.31  (0.87, 1.98)  P=0.193 | 0.98  (0.58, 1.67)  P=0.946 | 1.06  (0.70, 1.60)  P=0.778 |
| Ciprofloxacin | **24.02**  **(18.85, 30.60)**  **P<0.001** | **10.41**  **(7.33, 14.76)**  **P<0.001** | **13.27**  **(8.11, 21.70)**  **P<0.001** | **3.85**  **(2.66, 5.57)**  **P<0.001** |
| Nitrofurantoin | 1.35  (0.98, 1.85)  P=0.063 | 1.24  (0.76, 2.04)  P=0.392 | 0.87  (0.47, 1.64)  P=0.673 | 0.73  (0.43, 1.25)  P=0.246 |
| Trimethoprim | 0.84  (0.66, 1.06)  P=0.136 | 1.10  (0.79, 1.53)  P=0.569 | 0.76  (0.48, 1.20)  P=0.230 | 1.25  (0.89, 1.76)  P=0.199 |

Note: Models control for AMR resistance to all antibiotics shown at the first infection and their interactions with inter-infection time, calendar year at second infection, and age at second infection. OR is odds ratio. CI is confidence interval. Pivmecillinam and fosfomycin were excluded from multi-antibiotic models as the numbers of resistant organisms and number of cultures reporting sensitivity results were low.

**Table S7:** Associations between **nitrofurantoin** resistance at second infection with resistance to all antibiotic of interest at first infection, by inter-infection time, controlling for all antibiotic susceptibilities at first infection (multi-antibiotic models).

|  | 0 to <3  months  OR (95% CI) | 3 to <6  months  OR (95% CI) | 6 to <12 months  OR (95% CI) | ≥12  Months  OR (95% CI) |
| --- | --- | --- | --- | --- |
| Amoxicillin | **1.50**  **(1.12, 2.02)**  **P=0.007** | 1.04  (0.67, 1.62)  P=0.866 | 1.30  (0.74, 2.31)  P=0.366 | 1.12  (0.72, 1.73)  P=0.616 |
| Co-Amoxiclav | 0.78  (0.55, 1.10)  P=0.152 | 1.12  (0.66, 1.89)  P=0.672 | 1.34  (0.70, 2.56)  P=0.384 | 0.76  (0.44, 1.30)  P=0.316 |
| Cefalexin | 1.29  (0.93, 1.78)  P=0.127 | 1.04  (0.65, 1.67)  P=0.866 | 1.55  (0.89, 2.70)  P=0.122 | **1.72**  **(1.10, 2.69)**  **P=0.017** |
| Ciprofloxacin | 0.85  (0.58, 1.23)  P=0.373 | 1.31  (0.77, 2.25)  P=0.320 | 0.85  (0.41, 1.78)  P=0.671 | 1.07  (0.63, 1.83)  P=0.800 |
| Nitrofurantoin | **14.02**  **(10.81, 18.18)**  **P<0.001** | **6.32**  **(4.13, 9.67)**  **P<0.001** | **5.10**  **(3.01, 8.66)**  **P<0.001** | **3.41**  **(2.18, 5.33)**  **P<0.001** |
| Trimethoprim | 1.23  (0.93, 1.61)  P=0.149 | 0.78  (0.51, 1.18)  P=0.239 | 1.05  (0.64, 1.75)  P=0.840 | 1.11  (0.74, 1.66)  P=0.624 |

Note: Models control for AMR resistance to all antibiotics shown at the first infection and their interactions with inter-infection time, calendar year at second infection, and age at second infection. OR is odds ratio. CI is confidence interval. Pivmecillinam and fosfomycin were excluded from multi-antibiotic models as the numbers of resistant organisms and number of cultures reporting sensitivity results were low.

**Table S8:** Associations between **trimethoprim** resistance at second infection with resistance to all antibiotic of interest at first infection, by inter-infection time, controlling for all antibiotic susceptibilities at first infection (multi-antibiotic models).

|  | 0 to <3  months  OR (95% CI) | 3 to <6  months  OR (95% CI) | 6 to <12 months  OR (95% CI) | ≥12  Months  OR (95% CI) |
| --- | --- | --- | --- | --- |
| Amoxicillin | 1.19  (0.98, 1.45)  P=0.080 | 1.13  (0.85, 1.51)  P=0.414 | **1.58**  **(1.10, 2.27)**  **P=0.013** | 1.19  (0.91, 1.57)  P=0.200 |
| Co-Amoxiclav | 1.19  (0.94, 1.50)  P=0.152 | 0.96  (0.69, 1.34)  P=0.815 | 1.03  (0.66, 1.58)  P=0.911 | 0.91  (0.65, 1.27)  P=0.583 |
| Cefalexin | 0.95  (0.76, 1.19)  P=0.658 | **1.66**  **(1.21, 2.29)**  **P=0.002** | 1.13  (0.76, 1.70)  P=0.540 | 0.93  (0.69, 1.25)  P=0.614 |
| Ciprofloxacin | 0.94  (0.75, 1.18)  P=0.589 | 1.08  (0.77, 1.52)  P=0.665 | 1.13  (0.72, 1.78)  P=0.590 | 1.17  (0.84, 1.63)  P=0.366 |
| Nitrofurantoin | 1.26  (0.98, 1.62)  P=0.078 | 0.95  (0.63, 1.43)  P=0.807 | 0.70  (0.43, 1.14)  P=0.146 | 1.16  (0.80, 1.68)  P=0.438 |
| Trimethoprim | **11.26**  **(9.45, 13.41)**  **P<0.001** | **6.16**  **(4.81, 7.88)**  **P<0.001** | **3.82**  **(2.76, 5.29)**  **P<0.001** | **2.10**  **(1.64, 2.68)**  **P<0.001** |

Note: Models control for AMR resistance to all antibiotics shown at the first infection and their interactions with inter-infection time, calendar year at second infection, and age at second infection. OR is odds ratio. CI is confidence interval. Pivmecillinam and fosfomycin were excluded from multi-antibiotic models as the numbers of resistant organisms and number of cultures reporting sensitivity results were low.

**Table S9:** Associations between **amoxicillin** resistance at second infection with resistance to all antibiotic of interest at first infection **in UTI pairs caused by *E. coli***, by inter-infection time, controlling for all antibiotic susceptibilities at first infection (multi-antibiotic models).

|  | 0 to <3  months  OR (95% CI) | 3 to <6  months  OR (95% CI) | 6 to <12 months  OR (95% CI) | ≥12  Months  OR (95% CI) |
| --- | --- | --- | --- | --- |
| Amoxicillin | **28.25**  **(20.68, 38.59)**  **P<0.001** | **13.78**  **(9.09, 20.88)**  **P<0.001** | **8.37**  **(5.04, 13.90)**  **P<0.001** | **2.64**  **(1.82, 3.83)**  **P<0.001** |
| Co-Amoxiclav | 1.25  (0.87, 1.80)  P=0.221 | 0.98  (0.63, 1.54)  P=0.943 | 1.55  (0.84, 2.87)  P=0.159 | 0.85  (0.55, 1.30)  P=0.448 |
| Cefalexin | **0.66**  **(0.46, 0.93)**  **P=0.017** | 1.13  (0.72, 1.77)  P=0.592 | **0.48**  **(0.27, 0.87)**  **P=0.016** | 1.34  (0.92, 1.95)  P=0.125 |
| Ciprofloxacin | 1.40  (0.94, 2.08)  P=0.097 | 1.06  (0.64, 1.75)  P=0.821 | 1.34  (0.68, 2.65)  P=0.404 | **1.87**  **(1.09, 3.19)**  **P=0.022** |
| Nitrofurantoin | 2.06  (0.83, 5.10)  P=0.120 | 1.25  (0.34, 4.62)  P=0.738 | 3.60  (0.89, 14.55)  P=0.072 | 1.94  (0.56, 6.73)  P=0.298 |
| Trimethoprim | 0.93  (0.72, 1.21)  P=0.583 | 0.74  (0.52, 1.04)  P=0.080 | 0.84  (0.53, 1.31)  P=0.085 | 0.76  (0.55, 1.04)  P=0.085 |

Note: Models control for AMR resistance to all antibiotics shown at the first infection and their interactions with inter-infection time, calendar year at second infection, and age at second infection. OR is odds ratio. CI is confidence interval. Pivmecillinam and fosfomycin were excluded from multi-antibiotic models as the numbers of resistant organisms and number of cultures reporting sensitivity results were low.

**Table S10:** Associations between **co-amoxiclav** resistance at second infection with resistance to all antibiotic of interest at first infection **in UTI pairs caused by *E. coli***, by inter-infection time, controlling for all antibiotic susceptibilities at first infection (multi-antibiotic models).

|  | 0 to <3  months  OR (95% CI) | 3 to <6  months  OR (95% CI) | 6 to <12 months  OR (95% CI) | ≥12  Months  OR (95% CI) |
| --- | --- | --- | --- | --- |
| Amoxicillin | **3.15**  **(2.38, 4.18)**  **P<0.001** | **2.66**  **(1.73, 4.10)**  **P<0.001** | **3.71**  **(2.21, 6.25)**  **P<0.001** | 1.22  (0.81, 1.84)  P=0.335 |
| Co-Amoxiclav | **5.97**  **(4.52, 7.90)**  **P<0.001** | **4.34**  **(2.84, 6.63)**  **P<0.001** | **2.56**  **(1.48, 4.45)**  **P<0.001** | **1.66**  **(1.05, 2.62)**  **P=0.030** |
| Cefalexin | 1.23  (0.93, 1.64)  P=0.151 | **1.55**  **(1.03, 2.33)**  **P=0.036** | 0.99  (0.58, 1.69)  P=0.959 | 1.21  (0.82, 1.78)  P=0.341 |
| Ciprofloxacin | 1.23  (0.89, 1.72)  P=0.211 | 0.92  (0.57, 1.48)  P=0.720 | 1.65  (0.87, 3.15)  P=0.128 | **2.20**  **(1.31, 3.69)**  **P=0.003** |
| Nitrofurantoin | 1.13  (0.54, 2.36)  P=0.753 | 2.92  (0.81, 10.54)  P=0.101 | 1.54  (0.40, 5.95)  P=0.529 | 1.82  (0.57, 5.81)  P=0.312 |
| Trimethoprim | 1.00  (0.80, 1.26)  P=0.980 | 0.92  (0.66, 1.28)  P=0.600 | 0.71  (0.45, 1.10)  P=0.125 | 0.86  (0.61, 1.20)  P=0.379 |

Note: Models control for AMR resistance to all antibiotics shown at the first infection and their interactions with inter-infection time, calendar year at second infection, and age at second infection. OR is odds ratio. CI is confidence interval. Pivmecillinam and fosfomycin were excluded from multi-antibiotic models as the numbers of resistant organisms and number of cultures reporting sensitivity results were low.

**Table S11:** Associations between **cefalexin** resistance at second infection with resistance to all antibiotic of interest at first infection **in UTI pairs caused by *E. coli***, by inter-infection time, controlling for all antibiotic susceptibilities at first infection (multi-antibiotic models).

|  | 0 to <3  months  OR (95% CI) | 3 to <6  months  OR (95% CI) | 6 to <12 months  OR (95% CI) | ≥12  Months  OR (95% CI) |
| --- | --- | --- | --- | --- |
| Amoxicillin | 1.17  (0.79, 1.72)  P=0.433 | 1.17  (0.65, 2.08)  P=0.603 | **2.95**  **(1.50, 5.79)**  **P=0.002** | 0.99  (0.55, 1.76)  P=0.963 |
| Co-Amoxiclav | 1.39  (0.94, 2.05)  P=0.103 | 0.99  (0.54, 1.81)  P=0.963 | 0.95  (0.46, 1.92)  P=0.875 | 1.41  (0.75, 2.64)  P=0.292 |
| Cefalexin | **10.86**  **(8.00, 14.73)**  **P<0.001** | **11.53**  **(7.00, 19.01)**  **P<0.001** | **3.05**  **(1.62, 5.76)**  **P<0.001** | **2.07**  **(1.26, 3.42)**  **P=0.004** |
| Ciprofloxacin | **1.68**  **(1.16, 2.43)**  **P=0.006** | 1.15  (0.66, 2.01)  P=0.627 | **3.12**  **(1.50, 6.52)**  **P=0.002** | **1.87**  **(1.02, 3.42)**  **P=0.044** |
| Nitrofurantoin | 0.92  (0.43, 1.98)  P=0.833 | 0.69  (0.14, 3.40)  P=0.651 | **5.85**  **(1.09, 31.56)**  **P=0.040** | 2.54  (0.76, 8.53)  P=0.132 |
| Trimethoprim | 1.09  (0.82, 1.44)  P=0.576 | 1.07  (0.70, 1.64)  P=0.758 | 0.66  (0.38, 1.16)  P=0.147 | 1.00  (0.64, 1.56)  P=0.994 |

Note: Models control for AMR resistance to all antibiotics shown at the first infection and their interactions with inter-infection time, calendar year at second infection, and age at second infection. OR is odds ratio. CI is confidence interval. Pivmecillinam and fosfomycin were excluded from multi-antibiotic models as the numbers of resistant organisms and number of cultures reporting sensitivity results were low.

**Table S12:** Associations between **ciprofloxacin** resistance at second infection with resistance to all antibiotic of interest at first infection **in UTI pairs caused by *E. coli***, by inter-infection time, controlling for all antibiotic susceptibilities at first infection (multi-antibiotic models).

|  | 0 to <3  months  OR (95% CI) | 3 to <6  months  OR (95% CI) | 6 to <12 months  OR (95% CI) | ≥12  Months  OR (95% CI) |
| --- | --- | --- | --- | --- |
| Amoxicillin | 1.48  (0.88, 2.52)  P=0.142 | 1.67  (0.89, 3.13)  P=0.110 | 1.68  (0.74, 3.78)  P=0.212 | 1.35  (0.71, 2.57)  P=0.361 |
| Co-Amoxiclav | 1.15  (0.67, 1.98)  P=0.620 | 0.67  (0.34, 1.31)  P=0.239 | 0.76  (0.30, 1.90)  P=0.551 | 0.72  (0.35, 1.49)  P=0.375 |
| Cefalexin | 0.82  (0.49, 1.37)  P=0.438 | 1.21  (0.63, 2.32)  P=0.574 | 1.13  (0.46, 2.82)  P=0.786 | 1.36  (0.71, 2.59)  P=0.350 |
| Ciprofloxacin | **98.9**  **(64.56, 151.56)**  **P<0.001** | **25.12**  **(14.73, 42.83)**  **P<0.001** | **31.50**  **(14.73, 67.38)**  **P<0.001** | **7.83**  **(4.31, 14.23)**  **P<0.001** |
| Nitrofurantoin | 2.16  (0.72, 6.43)  P=0.167 | 1.48  (0.32, 6.85)  P=0.618 | 0.98  (0.12, 8.11)  P=0.981 | 2.27  (0.57, 9.03)  P=0.244 |
| Trimethoprim | 0.96  (0.63, 1.48)  P=0.862 | 1.25  (0.76, 2.06)  P=0.380 | 1.02  (0.50, 2.09)  P=0.954 | 1.32  (0.78, 2.25)  P=0.305 |

Note: Models control for AMR resistance to all antibiotics shown at the first infection and their interactions with inter-infection time, calendar year at second infection, and age at second infection. OR is odds ratio. CI is confidence interval. Pivmecillinam and fosfomycin were excluded from multi-antibiotic models as the numbers of resistant organisms and number of cultures reporting sensitivity results were low.

**Table S13:** Associations between **nitrofurantoin** resistance at second infection with resistance to all antibiotic of interest at first infection **in UTI pairs caused by *E. coli***, by inter-infection time, controlling for all antibiotic susceptibilities at first infection (multi-antibiotic models).

|  | 0 to <3  months  OR (95% CI) | 3 to <6  months  OR (95% CI) | 6 to <12 months  OR (95% CI) | ≥12  Months  OR (95% CI) |
| --- | --- | --- | --- | --- |
| Amoxicillin | 1.25  (0.47, 3.30)  P=0.654 | 0.56  (0.06, 4.84)  P=0.594 | 6.37  (0.48, 85.38)  P=0.162 | 0.63  (0.17, 2.37)  P=0.494 |
| Co-Amoxiclav | 0.76  (0.29, 2.02)  P=0.779 | 2.07  (0.22, 19.13)  P=0.522 | 0.53  (0.09, 3.31)  P=0.498 | 2.15  (0.52, 8.80)  P=0.289 |
| Cefalexin | 0.68  (0.24, 1.91)  P=0.463 | 2.68  (0.67, 10.66)  P=0.163 | 1.75  (0.26, 11.98)  P=0.568 | 0.92  (0.31, 2.76)  P=0.882 |
| Ciprofloxacin | 0.66  (0.23, 1.92)  P=0.448 | 1.34  (0.33, 5.48)  P=0.687 | 0.79  (0.11, 5.52)  P=0.809 | 0.70  (0.14, 3.36)  P=0.651 |
| Nitrofurantoin | **107.81**  **(45.99, 252.75)**  **P<0.001** | **25.50**  **(6.49, 100.23)**  **P<0.001** | **34.02**  **(4.81, 240.92)**  **P<0.001** | **6.18**  **(1.18, 32.47)**  **P=0.030** |
| Trimethoprim | **2.88**  **(1.28, 6.50)**  **P=0.011** | 0.91  (0.27, 3.06)  P=0.883 | 5.08  (0.83, 31.16)  P=0.079 | 1.71  (0.70, 4.20)  P=0.239 |

Note: Models control for AMR resistance to all antibiotics shown at the first infection and their interactions with inter-infection time, calendar year at second infection, and age at second infection. OR is odds ratio. CI is confidence interval. Pivmecillinam and fosfomycin were excluded from multi-antibiotic models as the numbers of resistant organisms and number of cultures reporting sensitivity results were low.

**Table S14:** Associations between **trimethoprim** resistance at second infection with resistance to all antibiotic of interest at first infection **in UTI pairs caused by *E. coli***, by inter-infection time, controlling for all antibiotic susceptibilities at first infection (multi-antibiotic models).

|  | 0 to <3  months  OR (95% CI) | 3 to <6  months  OR (95% CI) | 6 to <12 months  OR (95% CI) | ≥12  Months  OR (95% CI) |
| --- | --- | --- | --- | --- |
| Amoxicillin | 1.00  (0.73, 1.38)  P=0.999 | 1.09  (0.71, 1.67)  P=0.686 | 1.42  (0.83, 2.42)  P=0.200 | 1.18  (0.80, 1.76)  P=0.406 |
| Co-Amoxiclav | 1.26  (0.88, 1.79)  P=0.202 | 0.74  (0.46, 1.17)  P=0.195 | 0.78  (0.43, 1.43)  P=0.424 | 0.66  (0.41, 1.05)  P<0.080 |
| Cefalexin | **0.58**  **(0.41, 0.83)**  **P=0.003** | **2.13**  **(1.35, 3.37)**  **P=0.001** | 1.24  (0.69, 2.24)  P=0.475 | 1.29  (0.86, 1.95)  P=0.220 |
| Ciprofloxacin | 1.05  (0.72, 1.54)  P=0.794 | 1.48  (0.89, 2.44)  P=0.128 | 1.23  (0.63, 2.41)  P=0.545 | 1.19  (0.70, 2.03)  P=0.514 |
| Nitrofurantoin | 2.17  (0.92, 5.08)  P=0.075 | 1.03  (0.27, 3.87)  P=0.970 | 0.75  (0.18, 3.13)  P=0.698 | 1.75  (0.56, 5.44)  P=0.335 |
| Trimethoprim | **33.49**  **(25.79, 43.49)**  **P<0.001** | **12.20**  **(8.74, 17.01)**  **P<0.001** | **7.79**  **(5.00, 12.15)**  **P<0.001** | **2.35**  **(1.69, 3.27)**  **P<0.001** |

Note: Models control for AMR resistance to all antibiotics shown at the first infection and their interactions with inter-infection time, calendar year at second infection, and age at second infection. OR is odds ratio. CI is confidence interval. Pivmecillinam and fosfomycin were excluded from multi-antibiotic models as the numbers of resistant organisms and number of cultures reporting sensitivity results were low.

**Table S15:** Association between antimicrobial resistance at first and second infection by inter-infection time for all bacterial species for eight antibiotics commonly used in primary care, **stratified by age.**

|  | 0 to <3  months  OR (95% CI) | 3 to <6  months  OR (95% CI) | 6 to <12 months  OR (95% CI) | ≥12  Months  OR (95% CI) |
| --- | --- | --- | --- | --- |
| Amoxicillin |  |  |  |  |
| < 50 Years | **13.32**  **(10.32, 17.18)**  **P<0.001** | **6.73**  **(4.70, 9.63)**  **P<0.001** | **4.26**  **(2.43, 7.46)**  **P<0.001** | **1.50**  **(1.00, 2.24)**  **P=0.048** |
| ≥ 50 Years | **12.00**  **(10.15, 14.20)**  **P<0.001** | **6.51**  **(5.17, 8.21)**  **P<0.001** | **5.02**  **(3.79, 6.63)**  **P<0.001** | **1.94**  **(1.58, 2.37)**  **P<0.001** |
| Co-Amoxiclav |  |  |  |  |
| < 50 Years | **12.18**  **(8.87, 16.74)**  **P<0.001** | **6.16**  **(3.91, 9.70)**  **P<0.001** | **3.21**  **(1.52, 6.81)**  **P=0.002** | 1.33  (0.77, 2.31)  P=0.314 |
| ≥ 50 Years | **10.14**  **(8.42, 12.22)**  **P<0.001** | **7.48**  **(5.71, 9.79)**  **P<0.001** | **4.63**  **(3.34, 6.41)**  **P<0.001** | **2.31**  **(1.80, 2.96)**  **P<0.001** |
| Cefalexin |  |  |  |  |
| < 50 Years | **13.60**  **(9.06, 20.42)**  **P<0.001** | **5.39**  **(2.97, 9.80)**  **P<0.001** | **6.06**  **(2.30, 15.94)**  **P<0.001** | 2.09  (0.98, 4.47)  P=0.057 |
| ≥ 50 Years | **8.79**  **(7.06, 10.93)**  **P<0.001** | **7.15**  **(5.10, 10.03)**  **P<0.001** | **3.75**  **(2.50, 5.61)**  **P<0.001** | **2.02**  **(1.49, 2.75)**  **P<0.001** |
| Ciprofloxacin |  |  |  |  |
| < 50 Years | **19.23**  **(13.91, 26.60)**  **P<0.001** | **12.26**  **(7.17, 20.98)**  **P<0.001** | **7.26**  **(3.26, 16.13)**  **P<0.001** | **2.01**  **(1.03, 3.95)**  **P=0.042** |
| ≥ 50 Years | **19.78**  **(15.65, 25.01)**  **P<0.001** | **8.88**  **(6.38, 12.37)**  **P<0.001** | **8.15**  **(5.32, 12.47)**  **P<0.001** | **4.29**  **(3.04, 6.05)**  **P<0.001** |
| Fosfomycin |  |  |  |  |
| < 50 Years | **23.29**  **(10.57, 51.31)**  **P<0.001** | **30.52**  **(9.80, 95.01)**  **P<0.001** | **16.98**  **(2.54, 113.44)**  **P=0.003** | 2.11  (0.25, 17.85)  P=0.493 |
| ≥ 50 Years | **17.76**  **(11.55, 27.30)**  **P<0.001** | **8.81**  **(4.24, 18.30)**  **P<0.001** | **13.73**  **(5.10, 37.00)**  **P<0.001** | **2.55**  **(1.11, 5.86)**  **P=0.027** |
| Nitrofurantoin |  |  |  |  |
| < 50 Years | **21.88**  **(13.02, 36.76)**  **P<0.001** | **11.46**  **(5.13, 25.63)**  **P<0.001** | **16.86**  **(5.07, 56.08)**  **P<0.001** | 2.43  (0.67, 8.80)  P=0.177 |
| ≥ 50 Years | **13.58**  **(10.46, 17.62)**  **P<0.001** | **5.42**  **(3.52, 8.34)**  **P<0.001** | **4.82**  **(2.88, 8.07)**  **P<0.001** | **3.91**  **(2.59, 5.90)**  **P<0.001** |
| Pivmecillinam |  |  |  |  |
| < 50 Years | **58.53**  **(24.01, 142.68)**  **P<0.001** | **28.21**  **(8.24, 96.60)**  **P<0.001** | * | 2.49  (0.23, 27.13  P=0.454 |
| ≥ 50 Years | **37.17**  **(23.45, 58.92)**  **P<0.001** | **13.76**  **(6.25, 30.29)**  **P<0.001** | **12.71**  **(4.41, 36.60)**  **P<0.001** | **5.33**  **(2.12, 13.39)**  **P<0.001** |
| Trimethoprim |  |  |  |  |
| < 50 Years | **9.60**  **(7.49, 12.31)**  **P<0.001** | **5.87**  **(4.07, 8.47)**  **P<0.001** | **2.66**  **(1.44, 4.93)**  **P=0.002** | **1.73**  **(1.12, 2.67)**  **P=0.013** |
| ≥ 50 Years | **11.63**  **(9.77, 13.85)**  **P<0.001** | **6.00**  **(4.71, 7.65)**  **P<0.001** | **5.29**  **(3.92, 7.12)**  **P<0.001** | **2.43**  **(1.94, 3.04)**  **P<0.001** |

Note: All (single-antibiotic) models control for calendar year at second infection, and age at second infection. OR is odds ratio. CI is confidence interval. *model did not converge due to sparse cell counts

**Table S16:** Association between antimicrobial resistance at first and second infection in the 0 to <3 month time window for all bacterial species for eight antibiotics commonly used in primary care, **comparing a 14 day definition of independent infections to a 28 day sensitivity analysis definition of independent infections.**

|  | 14 day definition  (main models)  OR (95% CI) | 28 day definition  (sensitivity analysis)  OR (95% CI) |
| --- | --- | --- |
| Amoxicillin | **12.38**  **(10.76, 14.25)**  **p<0.001** | **10.50**  **(8.94, 12.33)**  **p<0.001** |
| Co-Amoxiclav | **10.64**  **(9.06, 12.49)**  **p<0.001** | **9.28**  **(7.70, 11.19)**  **p<0.001** |
| Cefalexin | **9.75**  **(8.04, 11.82)**  **p<0.001** | **9.11**  **(7.27, 11.42)**  **p<0.001** |
| Ciprofloxacin | **19.65**  **(16.30, 23.75)**  **p<0.001** | **16.62**  **(13.29, 20.77)**  **p<0.001** |
| Fosfomycin | **19.90**  **(13.66, 28.92)**  **p<0.001** | **18.27**  **(11.98, 27.87)**  **p<0.001** |
| Nitrofurantoin | **15.04**  **(11.91, 19.00)**  **p<0.001** | **13.71**  **(10.46, 17.97)**  **p<0.001** |
| Pivmecillinam | **41.70**  **(27.70, 62.80)**  **p<0.001** | **37.60**  **(23.24, 60.83)**  **p<0.001** |
| Trimethoprim | **10.94**  **(9.48, 12.61)**  **p<0.001** | **9.38**  **(7.95, 11.06)**  **p<0.001** |

Note: All (single-antibiotic) models control for calendar year at second infection, and age at second infection. OR is odds ratio. CI is confidence interval.
